# Substitution Models of Protein Evolution with Selection on Enzymatic Activity

**DOI:** 10.1093/molbev/msae026

**Published:** 2024-02-05

**Authors:** David Ferreiro, Ruqaiya Khalil, Sergio F Sousa, Miguel Arenas

**Affiliations:** CINBIO, Universidade de Vigo, 36310 Vigo, Spain; Department of Biochemistry, Genetics and Immunology, Universidade de Vigo, 36310 Vigo, Spain; CINBIO, Universidade de Vigo, 36310 Vigo, Spain; Department of Biochemistry, Genetics and Immunology, Universidade de Vigo, 36310 Vigo, Spain; UCIBIO/REQUIMTE, BioSIM, Departamento de Biomedicina, Faculdade de Medicina da Universidade do Porto, 4200-319 Porto, Portugal; CINBIO, Universidade de Vigo, 36310 Vigo, Spain; Department of Biochemistry, Genetics and Immunology, Universidade de Vigo, 36310 Vigo, Spain

**Keywords:** substitution model, protein evolution, molecular evolution, protein function, molecular dynamics simulations, protein phylogenetics

## Abstract

Substitution models of evolution are necessary for diverse evolutionary analyses including phylogenetic tree and ancestral sequence reconstructions. At the protein level, empirical substitution models are traditionally used due to their simplicity, but they ignore the variability of substitution patterns among protein sites. Next, in order to improve the realism of the modeling of protein evolution, a series of structurally constrained substitution models were presented, but still they usually ignore constraints on the protein activity. Here, we present a substitution model of protein evolution with selection on both protein structure and enzymatic activity, and that can be applied to phylogenetics. In particular, the model considers the binding affinity of the enzyme–substrate complex as well as structural constraints that include the flexibility of structural flaps, hydrogen bonds, amino acids backbone radius of gyration, and solvent-accessible surface area that are quantified through molecular dynamics simulations. We applied the model to the HIV-1 protease and evaluated it by phylogenetic likelihood in comparison with the best-fitting empirical substitution model and a structurally constrained substitution model that ignores the enzymatic activity. We found that accounting for selection on the protein activity improves the fitting of the modeled functional regions with the real observations, especially in data with high molecular identity, which recommends considering constraints on the protein activity in the development of substitution models of evolution.

## Introduction

Substitution models of evolution are routinely used in diverse evolutionary analyses of molecular data such as phylogenetic tree and ancestral sequence reconstructions (Arenas and Bastolla 2020; Furukawa et al. 2020; Arenas 2022; Ferreiro et al. 2022; Moreira et al. 2023), estimation of evolutionary parameters (Duchêne et al. 2016; Ghafari et al. 2021; Arenas 2021), and detection of genetic signatures of molecular adaptation (Yang and Nielsen 2002; Kosakovsky Pond and Frost 2005), among others. A large variety of substitution models are currently available (Arenas 2015a), and it is known that the accuracy of the evolutionary inferences is affected by the used substitution model (Lemmon and Moriarty 2004; Duchêne et al. 2016; Del Amparo and Arenas 2022a, 2023). Therefore, identifying and applying an appropriate substitution model of evolution is convenient to obtain as realistic as possible evolutionary inferences (Darriba et al. 2011; Kalyaanamoorthy et al. 2017).

At the protein level, empirical substitution models have been largely used in phylogenetics due to the relatively straightforward calculation of their substitution rates matrix (relative rates of change among amino acids) and amino acid frequencies at the equilibrium from a large amount of protein sequences (Yang 2007; Minh et al. 2021). However, empirical substitution models consider that all the protein sites evolve under the same substitution rates matrix and amino acid frequencies, which is an unrealistic assumption because, in nature, substitution patterns often differ among protein regions and sites (Shakhnovich et al. 1996; Pupko et al. 2002; Echave et al. 2016; Starr and Thornton 2016; Olabode et al. 2017; Ferreiro et al. 2022). A next generation of substitution models of protein evolution is the structurally constrained substitution (SCS) models. First, SCS models grouped sites into classes according to the secondary structure (i.e. α-helix, β-sheet, turn, and coil) (Thorne et al. 1996; Goldman et al. 1998) and many models also considered certain site-specific physicochemical attributes such as hydrophobicity and solvent accessibility (i.e. exposed or buried) (Koshi and Goldstein 1995; Goldman et al. 1998). Thus, the secondary structure was applied in diverse evolutionary analyses including phylogenetic tree and ancestral protein reconstructions (Lai et al. 2020; Pandey and Braun 2020). Subsequent SCS models combined protein biophysics and population genetics theories to model protein evolution with selection on thermodynamic attributes such as folding stability (see the reviews Sella and Hirsh 2005; Liberles et al. 2012; Echave and Wilke 2017) and also included substitution rate variation among protein sites (Echave et al. 2016). In general, SCS models surpassed empirical substitution models in terms of phylogenetic likelihood, distribution of site-specific amino acid frequencies, and folding stability of the modeled proteins (Parisi and Echave 2001; Fornasari et al. 2002; Bastolla et al. 2006; Arenas et al. 2013, 2015; Bordner and Mittelmann 2014; Arenas and Bastolla 2020; Del Amparo et al. 2023).

However, SCS models often ignore (or consider in a minor extent) constraints on the protein activity despite the observed constraints of protein activity on protein evolution (Saito et al. 1997; Bartlett et al. 2002; Echave et al. 2016; Jack et al. 2016; Echave 2021). For example, catalytic sites in enzymes tend to be highly conserved over evolutionary time and, in other sites, this conservation generally decreases when increasing their distance to the catalytic region of the protein structure (Abriata et al. 2015; Jack et al. 2016; Ribeiro et al. 2020). Indeed, the structural flexibility and molecular interactions for accommodating the substrate in the active site are crucial to allow the protein activity (Bartlett et al. 2002; Mittal et al. 2012) and thus can induce selective pressures (Fay and Wu 2003). However, counterintuitively to our knowledge, only one SCS model directly considered constraints from the protein activity (Echave 2019). In particular, that SCS model predicts the protein folding free energy and the activation energy barrier of the enzymatic reaction through an elastic network model that includes the entropy of the system (Echave 2008). Unfortunately, along with many other SCS models, the cited model was not implemented in a practical framework that would be useful for subsequent evaluations and evolutionary applications. Although one could expect constraints of the protein evolution caused by the protein activity, they should be formally investigated and implemented in more realistic and practical substitution models of evolution.

Here, we present a SCS model of protein evolution that considers the enzymatic activity (hereafter, the structure and activity constrained substitution [SACS] model) and we built and evaluated it with the HIV-1 protease (PR). The SACS model takes into account the binding-free energy landscape of the enzyme complexed with a common natural substrate, by long molecular dynamics (MD) simulations and the molecular mechanics–generalized Born surface area, in all the possible variants derived from site-specific substitution events. The model also includes structural parameters that inform about the stability and activity of the enzyme such as enzyme–substrate hydrophobic and polar contacts, flexibility of the PR flaps conformations, solvent accessibility, and amino acid backbone radius of gyration (RG), again evaluated for all the variants derived from site-specific substitution events. The SACS model provides site-specific substitution rates matrices and amino acid frequencies that can be used in phylogenetic methods based on maximum likelihood. We compared the SACS model with selected empirical substitution models and an SCS model that ignores the protein activity by their fitting with diverse HIV-1 PR real data.

## Materials and Methods

### Data for Building the SACS Model for the HIV-1 PR

We selected an X-ray structure of the HIV-1 PR with the consensus sequence of the HIV-1 Drug Resistance Database (HIVdb) (Shafer 2006) presenting the mutation D25N (required for preventing the catalysis) and a common natural substrate (PDB code 1KJH) (Prabu-Jeyabalan et al. 2002). In order to obtain the protein structure in the native state and natural conditions, that mutation was reverted (N25D) and the Asp25 was monoprotonated following previous studies (Arenas et al. 2009; McGee et al. 2014). The resulting protein structure was considered as a template (hereafter WT) for subsequent methods. Next, we obtained the PR sequence and structure variants derived from applying all the possible substitution events at each site of both chains. In the case of histidine, the two protonation states (Hid and Hie) were studied. Therefore, we obtained a total of 1,981 protein variants (99 sites × 20 amino acids that include 2 states for histidine and the WT variant) whose structures were structurally accommodated by MD simulations.

### MD Simulations

#### Protocol of MD Simulations to Analyze the Molecular Variants

We performed MD simulations with *AMBER22* (Case et al. 2022) to facilitate the structural accommodation of the WT and every variant. The systems for the MD simulations were prepared with the ff14SB force field (Maier et al. 2015). Indeed, they were solvated using a TIP3P water model (Jorgensen et al. 1983) in an orthogonal box with 12.0 Å of minimum distance and neutralized by counter ions with *Leap* (Case et al. 2022). Next, the solvated structures were refined through 4 consecutive minimization protocols, which consider nonbonded interactions with a cutoff of 10 Å, with *AMBER22* (PMEMD.cuda module) under the ff14SB force field. First, the water molecules and substituted residues (if any) were restrained by 50 kcal/mol/Å^2^ harmonic forces in 2,500 minimization steps (1,250 with the steepest descent method and 1,250 with the conjugated gradient method). Second, we used the same number and type of steps but with constraints only applied to hydrogen atoms. Next, also with the same number and type of steps, the constraints were applied to all the atoms except the backbone alfa carbon and nitrogen atoms. Finally, we conducted a global energy minimization based on 10,000 steps (5,000 with the steepest descent method and 5,000 with the conjugated gradient method).

We performed the MD simulations under periodic boundary conditions to simulate a continuous system. In particular, we used the SHAKE algorithm to fix all the bond lengths involving hydrogen atoms (Ryckaert et al. 1977) and an integration step of 2 fs. Overall, the MD simulation procedure included an equilibration procedure of 50 ps with constant number of particles, volume, and temperature (NVT) and a production protocol of 20 ns with constant number of particles, pressure, and temperature (NPT) according to the evaluations described in the following section. Indeed, we analyzed the resulting MD trajectories every 20 ps (1,000 frames), according to the following section, to predict structural properties such as average backbone root mean square deviation (RMSD), substitution backbone RG and solvent-accessible surface area (SASA), flap conformation as a function of the distance between catalytic sites (residues 25, 25′, 50, and 50′) and the hydrogen bond (HB) occupancy. We performed these analysis with the *CPPTRAJ* module (Roe and Cheatham 2013) implemented in *AMBER22TOOLS* (Case et al. 2022).

#### Evaluation of the MD Simulations at Variable Simulation Length and Trajectory Sampling

**Fig. 1.**
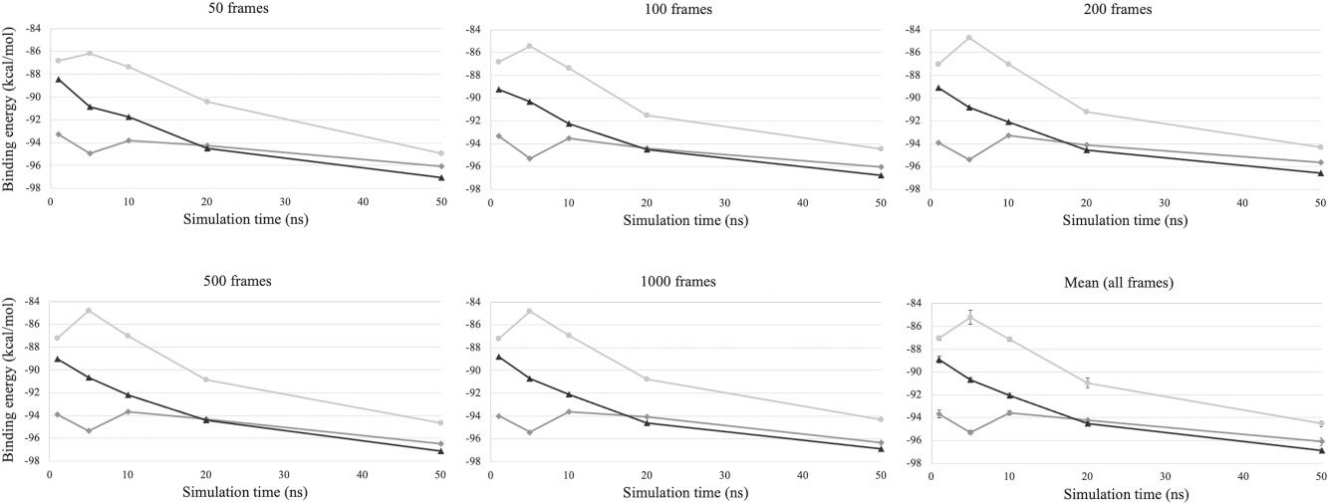
Predicted binding-free energy of WT HIV-1 PR with its natural substrate under variable MD simulation time and trajectory sampled frames. Binding-free energies of 3 replicates from MD simulations under a variety of trajectory sampled frames (50, 100, 200, 500, and 1,000 frames) and production times of simulation (1, 5, 10, 20, and 50 ns). It includes the mean of binding-free energies from the frames of each replicate at every simulation time. The error bars correspond to the 95% confidence interval from the mean.

We evaluated the influence of the simulation length (time) and trajectory sampling (number of frames) in MD simulations required to obtain accurate binding-free energy predictions under the WT structure. In particular, we performed 3 independent MD simulations and predicted the binding-free energy (see previous section for details) at 1, 5, 10, 20, and 50 ns of production run with variable recording frequencies (50, 100, 200, 500, and 1,000 frames). We found fluctuations of the binding-free energy in the shorter runs (1, 5, and 10 ns) regardless of the number of frames recorded in each run (Fig. 1; supplementary table S1, Supplementary Material online). In case of longer runs (20 and 50 ns), we obtained little variation of the binding-free energy regardless of the number of specified frames (Fig. 1 and supplementary table S1, Supplementary Material online). Therefore, considering the accuracy of the predictions and computational requirements, we selected trajectories of 20 ns long and a sampling scheme of 1,000 frames.

### Prediction of the Enzyme–Substrate Binding-Free Energies

The molecular mechanics–generalized Born surface area (MM-GBSA) (Genheden and Ryde 2015) can provide reliable predictions of the binding-free energy (*ΔG*) for diverse complexes such as protein–substrate, protein–inhibitor, and protein–protein (Ylilauri and Pentikäinen 2013; Chen et al. 2016; Weng et al. 2019), with acceptable computing times (Sun et al. 2014a, b; Forouzesh and Mishra 2021; Yu et al. 2022). The method considers van der Waals interactions, electrostatic interactions, polar and nonpolar solvation, and entropy, among other physicochemical parameters (Genheden and Ryde 2015). We used MM-GBSA, implemented in *AMBER22* (MMGBSA.py) (Miller et al. 2012; Case et al. 2022), to obtain the binding-free energy between the enzyme and the natural substrate. We analyzed every structure (WT and mutated variants), previously obtained with the MD simulations based on 1,000 frames, using the GB^OBC1^ model (Onufriev et al. 2004) and a salt concentration of 0.1 mol dm^−3^ (Miller et al. 2012).

### Prediction of the Protein Fitness Considering the Enzymatic Activity and Its Application to the Development of Substitution Models of Protein Evolution

We applied enzyme–substrate binding properties previously predicted for all the protein variants to derive the site-specific amino acid frequencies and substitution rates matrices (relative rates of change among amino acids) that are required by current likelihood-based phylogenetic methods. First, we calculated several physicochemical parameters related with the enzymatic activity for each protein variant obtained from all the possible amino acid (*i*) substitutions at each protein site (*s*). These parameters included the protein–substrate binding-free energy (*ΔG*), the size of catalytic box (Cdist), the RG, the SASA, and the HB occupancy (as indicated in the previous section). The selection of these parameters was based on two criteria; they are well established in the field for evaluating the stability of protein–substrate complexes (i.e. Cdist (Perryman et al. 2004; Karnati and Wang 2019), RG (Flores and Gerstein 2011; Yu et al. 2022), SASA (Porto et al. 2005; Bordner and Mittelmann 2014; Dubreuil and Levy 2021), and HB (Salentin et al. 2014; Gopal et al. 2017)) and can quantify the consequences of amino acid substitutions at least at sites related to the stability of the protein–substrate complex (details presented later). We obtained the site-specific weighted average of these parameters (Δ*G*, Cdist, RG, SASA, and HB) for the protein variants present in the query multiple sequence alignment. Next, we calculated the site-specific variation of the parameters (*ΔΔG*, *Δ*Cdist, *Δ*RG, *Δ*SASA, and *Δ*HB), between every amino acid and the corresponding weighted average in the protein variants of the query alignment, and then, we normalized the site-specific variation of the parameters with the sum of the variations. To further determine the influence of every amino acid at every site on the global stability of the corresponding enzyme–substrate system, we defined a factor *P* as a sum of the normalized variations of the mentioned parameters among amino acids at every site (Equation 1), normalized to sum 1. For an additional evaluation of the sensibility of the parameters of factor *P* to amino acid substitution events, we calculated the sum of every parameter for each protein variant derived from all the possible substitution events at sites 8 and 29 (involved in the protein–substrate binding) and 18 (located at the protein surface). We found that amino acid substitutions at site 18 cause minor variations of the factor *P* while substitutions at sites 8 and 29 produce larger variations (supplementary fig. S1, Supplementary Material online). Next, we defined a factor *K* that combines the global stability *P* with the enzyme–substrate affinity (binding-free energy, ΔΔG) (Equation 2). Thus, *K* provides information about the stability and activity of the enzyme–substrate system.


(1) $$P_{s,i}=|\Delta \mathrm{Cdis}t_{s,i}|+|\Delta RG_{s,i}|+|\Delta \mathrm{SAS}A_{s,i}|+|\Delta HB_{s,i}|$$

(2) $$K_{s,i}=|\Delta \Delta G_{s,i}|\times (1+(1\;\text{–}\;P_{s,i}))$$

Next, we calculated the protein fitness (*W*) for every variant with amino acid (*i*) at every site (*s*) by adapting the fitness function presented by Goldstein (2013) (Equation 3).


(3) $$W_{s,i}=\frac{1}{e^{K_{s,i}}}$$

Next, for every site, the fitness of the variants (derived from placing every amino acid *i* at that site *s*) was normalized considering the fitness of all the variants $\left(W_{s,i}=\frac{W_{s,i}}{\sum_{i}\;W_{s,i}}\right)$. Thus, the resulting normalized fitness can inform about the frequency of every amino acid at every site (producing a particular variant) (*f_s,i_*) by considering that variants with amino acids with a high fitness are usually more frequent due to selection (i.e. conservation of catalytic sites (Abriata et al. 2015; Jack et al. 2016; Ribeiro et al. 2020)). Indeed, it is well established that the protein structure affects both the rate of evolution and the observed amino acid frequencies (Overington et al. 1992; Goldman et al. 1998; Ramsey et al. 2011). Next, we calculated a site-specific symmetric substitution rates matrix that is required for inferences with common likelihood-based phylogenetic methods. First, we defined the relative rates of change between amino acids of an asymmetric substitution rates matrix *A_s_* as the difference of the fitness of the variants with those amino acids (Equation 4). Next, we calculated the site-specific symmetric substitution rates (*S_s,ij_*) (Equation 5) following Arenas et al. (2015) and satisfying detailed balance, which is required for current likelihood functions that assume site-independent modeling of molecular evolution. In this regard, we adapted the HIV-1 PR empirical substitution model (HIVpr, $E_{ij}^{\mathrm{emp}}$) (Del Amparo and Arenas 2022b) that best-fitted with the query data (according to Akaike and Bayesian Information Criteria, AIC and BIC, respectively) compared with other empirical substitution models (Del Amparo and Arenas 2022b). Thus, following previous studies on the development of SCS models with affordable computation (i.e. Arenas et al. 2015; Jimenez et al. 2018; Jiménez-Santos et al. 2018; Perron et al. 2019), we considered an empirical matrix for calibrating the global flux of relative rates of change among amino acids.


(4) $$A_{s}=|W_{s,i}\;-\;W_{s,j}|$$

(5) $$S_{s,ij}=E_{ij}^{\mathrm{emp}}\times \frac{\;f_{s,i}A_{s,ij}\;+\;f_{s,j}A_{s,ji}}{2f_{s,i}\;f_{s,j}}$$

### Evaluation of the SACS Model

We compared the SACS model with an empirical substitution model that was previously selected as the best-fitting empirical substitution model for the study data, the HIVpr empirical substitution model (used in Equation 5), and a SCS model that ignores constraints on the enzymatic activity, by phylogenetic likelihood with the study data.

We performed this evaluation with HIV-1 PR sequences available from GenBank that were also used as test data in a previous study (Del Amparo and Arenas 2022b). Importantly, the method data (used to build the models) and test data (used to evaluate the models) are independent, without sharing sequences. We excluded sequences with indels because the evaluated substitution models do not incorporate the modeling of indel evolution (empirical substitution models usually ignore indels and the SCS models cannot deal with indels in the protein structure). The data set presented a total of 1,271 protein sequences that were aligned with *Muscle* (Edgar 2004). The resulting multiple sequence alignment was partitioned into several data sets to study the influence of the molecular diversity on the performance of the substitution models and to reduce the computational burden. In particular, we built 9 data sets of 50 sequences each, where 3 data sets included randomly selected sequences with high similarity (average pairwise sequence identity 0.933, 0.933, and 0.934), other 3 data sets that included randomly selected sequences with intermediate level of similarity (average pairwise sequence identity 0.860, 0.867, and 0.864), and 3 data sets that included randomly selected sequences with low similarity (average pairwise sequence identity 0.787, 0.783, and 0.781).

Indeed, we analyzed 3 additional test data sets: in particular, an additional data set 1 that included 100 randomly selected HIV-1 PR sequences collected from naïve patients of the HIVdb and studied in Arenas (2015b); an additional data set 2 that included 113 HIV-1 PR sequences, also from naïve patients, of the HIVdb and studied in Ferreiro et al. (2022); and an additional data set 3 that included 95 retroviral PR sequences obtained from the PROSITE database (PS50175) (Sigrist et al. 2012). For every data set, we realigned the sequences with *Muscle*.

We found that the best-fitting empirical substitution model of protein evolution selected with *ModelTest-NG* (Darriba et al. 2020), among all the substitution models of protein evolution implemented in the framework, was, as expected, the HIVb substitution model (Nickle et al. 2007), and therefore, we compared the SACS models with this selected empirical substitution model and with the HIVpr empirical substitution model. In addition, we compared the SACS model with a SCS model. In particular, we used the mean-field (MF) SCS model (Minning et al. 2013; Arenas et al. 2015), which provides site-specific substitution rates matrices and amino acid frequencies at the equilibrium with constraints on the stability of the native state of the protein structure against both unfolded and misfolded states. Interestingly, previous studies showed that the MF model can produce a phylogenetic likelihood higher than that obtained with empirical and other SCS models when fitting diverse real protein families (Arenas et al. 2015). A common assumption in SCS models is that if the stability holds, then the activity does too (Bloom et al. 2006), and thus those models (including the MF model) ignore direct evolutionary constraints on the activity (Bordner and Mittelmann 2014; Arenas et al. 2015). Importantly, the MF model requires a representative protein structure of the test data set that we obtained by homology modeling with *SWISS-MODEL* (Arnold et al. 2006) considering as template the consensus sequence of the data set. Finally, we used *RAxML-NG* (Kozlov et al. 2019) to obtain the phylogenetic likelihood and the corresponding AIC and BIC scores (according to Luo et al. (2010)) by considering the number of parameters of the model at every branch of the derived ML phylogenetic tree and of the fitting of every substitution model with every test data set. Concerning the AIC and BIC scores, since the SACS and MF models infer the substitution rates matrix and the amino acid frequencies from empirical data (protein structure), as in the case of the empirical substitution model where they are inferred from protein sequences, we considered that these models share the same number of parameters. Additionally, we analyzed the fitting of each substitution model accounting for variable global substitution rate among protein sites according to a gamma distribution (+G) (Yang 1994, 1996) with each test data set, where the alpha shape parameter was estimated with *RAxML-NG*.

Finally, we evaluated the site-specific amino acid variability produced by the HIVb, HIVpr, MF, and SACS models in comparison with that observed in the test and additional test data (in both cases, all the study data sets were set together to consider more information) by the calculation of the effective number of amino acids *n*_eff_ (Equation 6), which is based on the sequence entropy *H* (Equation 7) (Strait and Dewey 1996; Goldstein and Pollock 2016; Echave and Wilke 2017; Johnson and Wilke 2020).


(6) $$n_{\mathrm{eff}}=e^{H}$$

(7) $$H=-\underset{i}{\sum }\;f_{i}\ln\;(\;f_{i})$$

## Results

### Validation of the SACS Model Based on Structural Information

#### Evaluation of the Predicted Enzyme–Substrate Binding at Illustrative Sites

As a control procedure, we evaluated the power of the method to predict the binding affinity of the WT and mutated enzyme variants with a natural substrate of the HIV-1 PR. In particular, we analyzed the prediction of the binding-free energy in variants derived from all the possible substitution events at sites 8 and 29 (directly involved in the protein–substrate binding) and at site 18 (located in the protein surface) to identify the influence of amino acid change in those sites on the binding-free energy. This evaluation provided a first overview on the power of the used MD protocol to detect the effect of substitution events at different sites on the stability of the enzyme–substrate complex. Substitutions at site 18 showed minor influences on the binding-free energy (supplementary fig. S2, Supplementary Material online), which was expected because this site is not directly involved in the binding with the substrate. In contrast, in both sites 8 and 29, the WT amino acid produced the most stable enzyme–substrate complex (supplementary fig. S2, Supplementary Material online), which could be expected because these sites are directly involved in the enzyme–substrate interaction through hydrogen bounds. In particular, the WT amino acid at those sites produced enzyme–substrate complexes with stability significantly higher than those for complexes based on most of the other amino acid states (supplementary fig. S2, Supplementary Material online). These results suggested that the used MD protocol can provide useful information about the consequences of substitution events occurring at sites directly related to the protein function.

#### Influence of Site-Specific Amino Acid Substitutions on the Enzyme–Substrate Binding

**Fig. 2.**
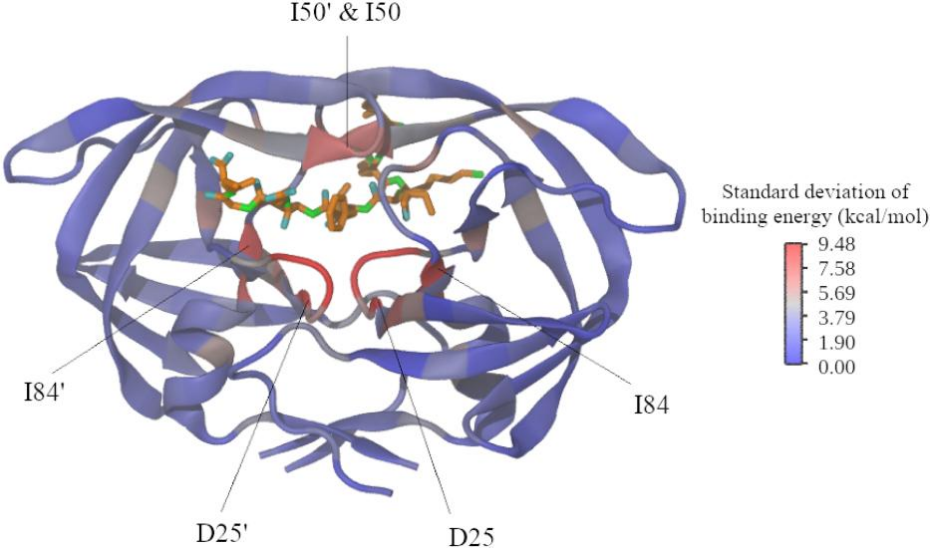
Variation of the binding-free energy of the HIV-1 PR with its natural substrate among protein variants derived from all possible site-specific amino acid substitutions. We evaluated the substrate–enzyme binding-free energy for molecular variants derived from all possible site-specific amino acid substitutions. The illustration shows the standard deviation from the predicted binding-free energies of the molecular variants. Thus, a high standard deviation at a site means that applying amino acid substitutions at that site dramatically affects the binding-free energy (the amino acid change affects the enzyme–substrate binding). Note that amino acid substitutions at sites of functional regions present a higher effect on the binding energy. A detailed representation of these site-specific standard deviations is provided in supplementary fig. S3, Supplementary Material online.

In agreement with the previous section, our predictions of the enzyme–substrate binding-free energy showed that substitution events at sites close to the substrate or involved in interactions with the substrate generally affect the enzyme–substrate binding (Fig. 2; supplementary fig. S3 and appendix, Supplementary Material online). In particular, we found that most of the substitution events in sites located at the protein core and flaps produced destabilization of the enzyme–substrate complex (increase of the binding-free energy), whereas substitution events in sites located at other enzyme regions (e.g. surface) barely affected the stability of the enzyme–substrate complex (supplementary figs. S2 and S3, Supplementary Material online).

We studied additional structural enzyme–substrate properties, obtained with MD simulations and included in the SACS model, that could provide information about effects of amino acid substitutions on enzyme–substrate interactions. First, as a control procedure, we calculated the RMSD between initial and modeled (derived from MD) protein conformations to observe the equilibrium of the system and structural fluctuations under the WT and the mutated variants. We found that amino acid substitutions produced protein variants with large equilibrium variability of the corresponding conformations (supplementary fig. S4, Supplementary Material online), which suggests a high robustness of the MD simulations in the studied trajectories. We also compared the enzyme–substrate HB occupancy between WT and each mutated variant through MD simulations (1,000 frames). We found that, as expected, protein variants derived from substitutions in sites close to the substrate, or involved in enzyme–substrate interactions, overall presented higher HB occupancy fluctuations compared with the WT variant (supplementary fig. S5 and appendix, Supplementary Material online). Next, we evaluated the distance between the sites 50, 50′, 25, and 25′ (catalytic box) to identify the influence of the different amino acid substitutions on the conformation of the PR flaps. The results showed that substitutions in sites located at the protein core (e.g. sites 25, 27, and 28) and flaps (e.g. sites 49 and 50) increase the size of the catalytic box (supplementary fig. S6 and appendix, Supplementary Material online). In contrast, we found that almost none substitution event produced changes of the RG, with the exception of residue 41 where all the variants except those with Arg (WT), Lys, and Tyr showed a significant variation of this parameter (supplementary fig. S7 and appendix, Supplementary Material online). In the same way, we found that only substitutions at site 1 produced major variations of SASA. In particular, any substitution of the WT amino acid (Pro) at that site broadly affected this parameter (supplementary fig. S8 and appendix, Supplementary Material online).

### Performance of the SACS Model

**Fig. 3.**
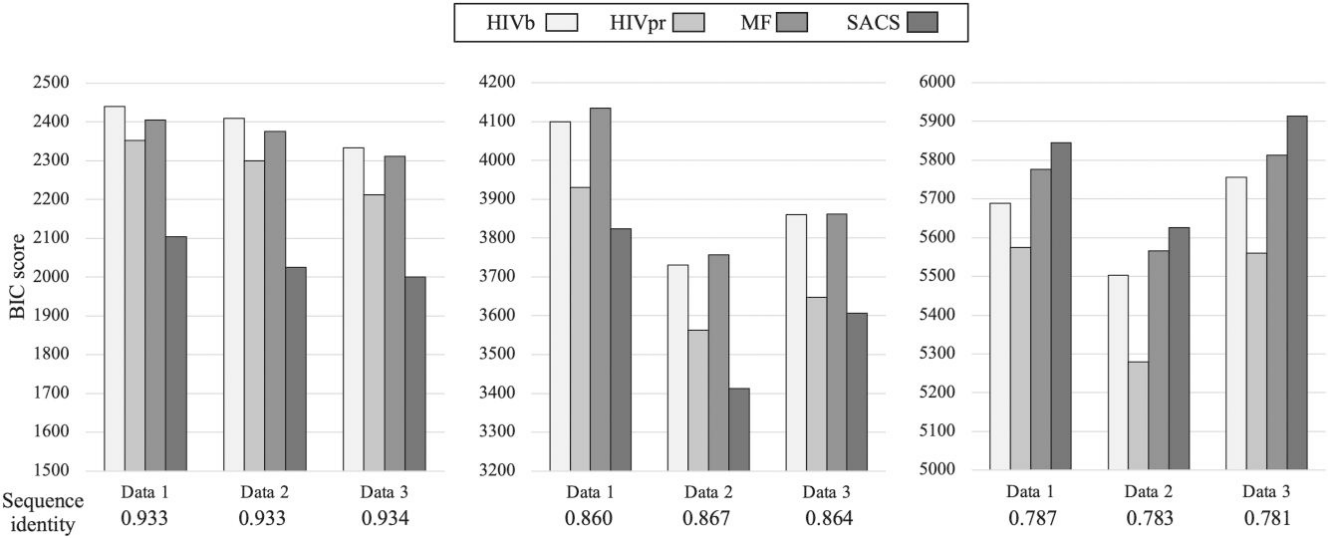
Fitting of the different substitution models of protein evolution with test data of different molecular diversities through phylogenetic likelihood. BIC scores obtained for the SACS model, the MF SCS model, the HIVb empirical substitution model (that was selected as the best-fitting empirical substitution model for the corresponding data among the empirical substitution models available in *ModelTest-NG*), and the HIVpr empirical substitution model, for several data with different sequence identity levels (scores for data with higher sequence identity are shown on the left, and scores for data with lower sequence identity are shown on the right). Note that a lower BIC score indicates a better fitting of that model with the data. The log-likelihood and AIC scores are presented in supplementary fig. S9, Supplementary Material online.

**Fig. 4.**
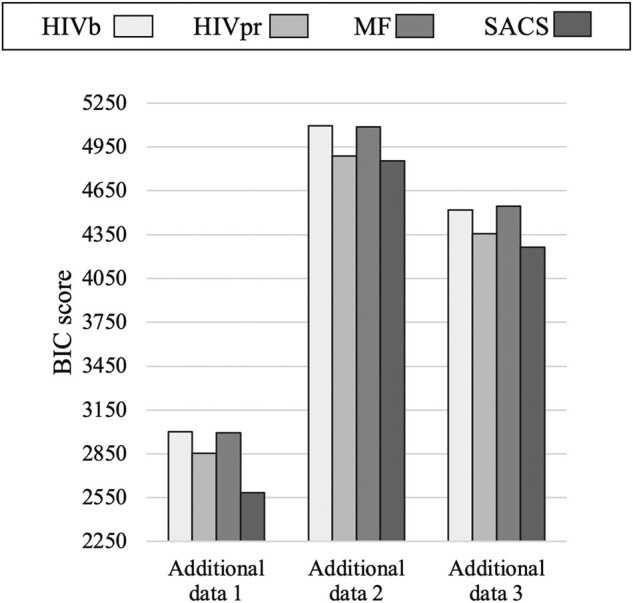
Fitting of the different substitution models of protein evolution with the additional test data through phylogenetic likelihood. BIC scores obtained for the SACS model, the MF SCS model, the HIVb empirical substitution model (that was selected as the best-fitting empirical substitution model for the corresponding data among the empirical substitution models available in *ModelTest-NG*), and the HIVpr empirical substitution model, for the 3 additional test data. Note that a lower BIC score indicates a better fitting of that model with the data. The log-likelihood and AIC scores are presented in supplementary fig. S11A, Supplementary Material online.

**Fig. 5.**
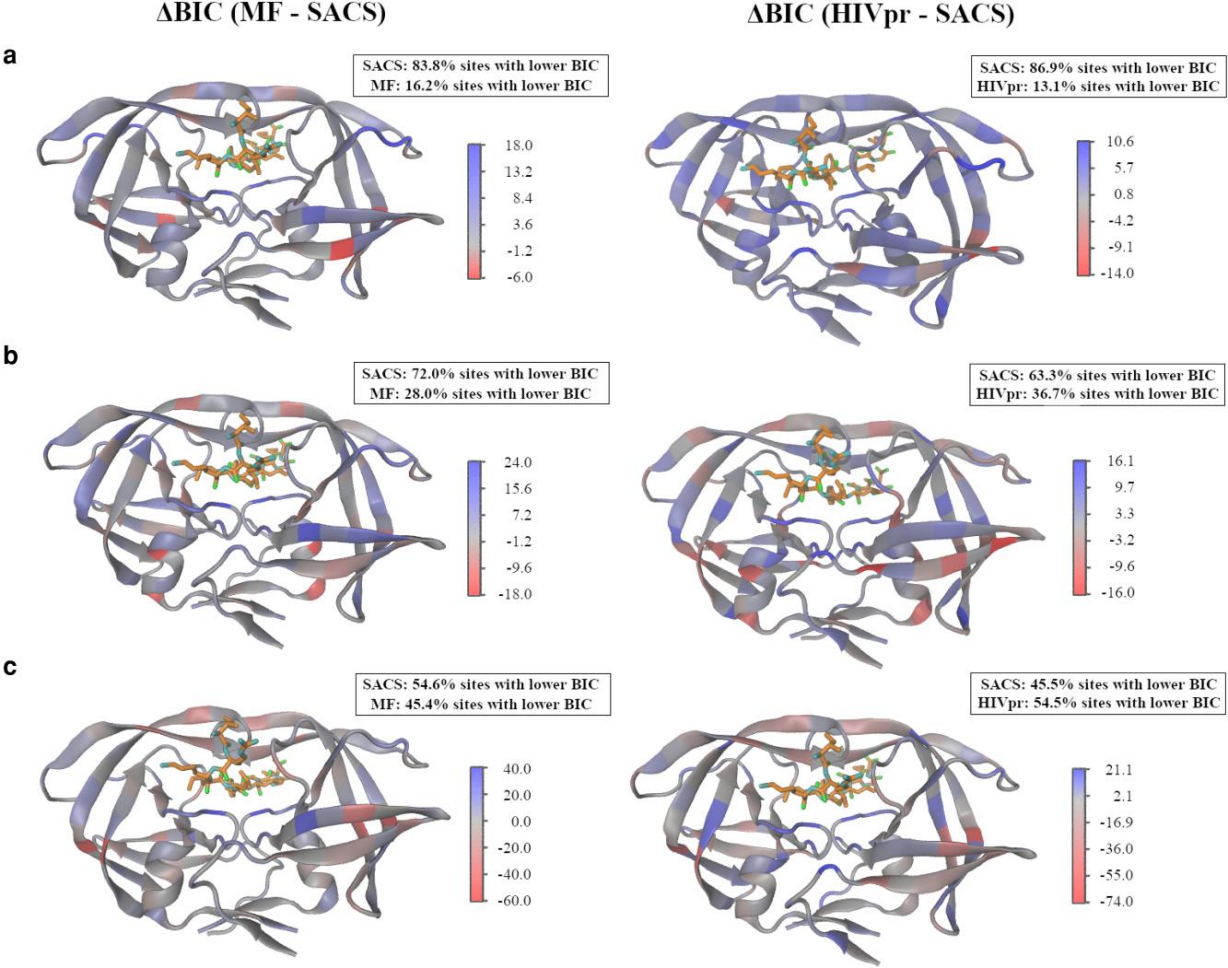
Site-specific fitting of the different substitution models of protein evolution with diverse test data through phylogenetic likelihood. The figure shows the difference in BIC scores between the MF SCS model and the SACS model (plots on the left) and between the HIVpr empirical substitution model and the SACS model (plots on the right); comparisons between the HIVb empirical substitution model (that was selected as the best-fitting empirical substitution model for the corresponding data among the empirical substitution models available in *ModelTest-NG*) and the SACS model are shown in supplementary fig. S14, Supplementary Material online (plots on the left). The analyses were performed for the data with high, intermediate, and low sequence identity (shown in panels a, b, and c, respectively). Note that positive ΔBIC scores indicate that the SACS model better fitted with the data at that site than the other substitution model. The boxes include the proportion of sites that were better fitted with any of the compared models. Further information about site-specific fitting of every studied model with the data is shown in supplementary table S2, Supplementary Material online.

We compared the performance of the SACS model with that from the MF, HIVb, and HIVpr substitution models in several test data sets with different sequence diversities. The results showed that the SACS model provides a better fitting (in terms of BIC and AIC scores) than the MF, HIVb, and HIVpr models in data sets with high and intermediate sequence identity (Fig. 3 and supplementary fig. S9, Supplementary Material online), while the HIVb and HIVpr substitution models were preferred for modeling the data with the lowest sequence identity (Fig. 3 and supplementary fig. S9, Supplementary Material online). We obtained similar trends when the 4 models consider variation of the global substitution rate among sites through a gamma distribution (supplementary fig. S10, Supplementary Material online). Regarding the analyses of the additional test data, we found that for the 3 additional data sets, the SACS model provided a better fitting than the other substitution models (Fig. 4 and supplementary fig. S11A, Supplementary Material online). Next, the site-specific fitting of every substitution model with the test data showed that the SACS model provides a better fitting in more protein sites at any level of sequence identity (77.1%, 52.9%, and 36.7% of sites for high, intermediate, and low sequence identity, respectively) than MF, HIVb, and HIVpr models (supplementary table S2 and fig. S12, Supplementary Material online; pairwise model comparisons represented on the protein structure are shown in Fig. 5). Therefore, the SACS model was preferred for more sites at any level of sequence identity, although especially for data with high sequence identity (supplementary table S2, Supplementary Material online), and it was not informative for some sites not directly related to the protein function (e.g. 62 and 73) (Fig. 5 and supplementary fig. S14, Supplementary Material online). Indeed, the site-specific analyses of the 3 additional data sets showed that the SACS model provided the best fitting with the data for most of the sites (supplementary table S3, Supplementary Material online). Considering variation of the global substitution rate among sites according to a gamma distribution in the models generally did not alter the previously presented outcomes, including the global fitting (supplementary figs. S10 and S11B, Supplementary Material online) and the site-specific fitting where most of the sites were better fitted by the SACS model (supplementary tables S2 and S3 and figs. S13 and S14, Supplementary Material online).

**Fig. 6.**
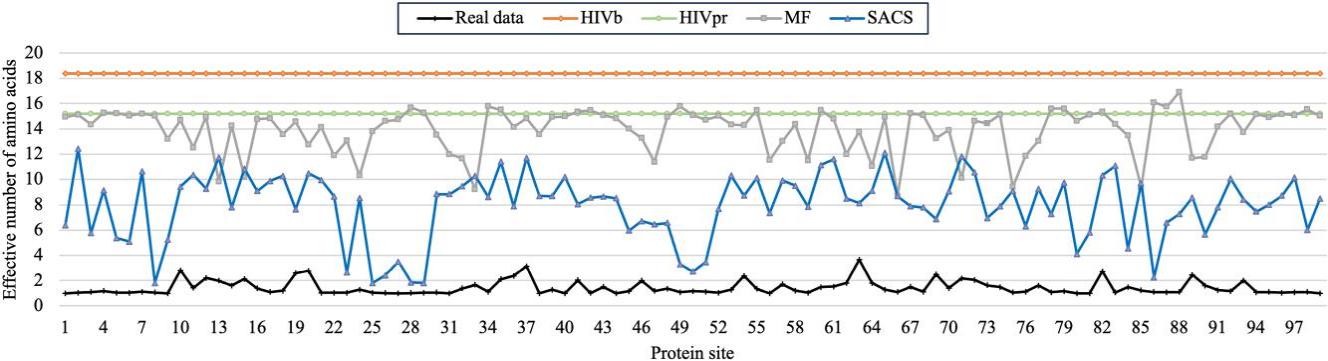
Site-specific effective number of amino acids in the test data and in the different substitution models of protein evolution. Site-specific effective number of amino acids obtained from all the real test data, the HIVb empirical substitution model, the HIVpr empirical substitution model, the MF SCS model, and the SACS model. Note that the most realistic substitution model is that one producing site-specific effective number of amino acids closer to those observed in the real data. Site-specific effective number of amino acids for the additional real test data are presented in supplementary fig. S15, Supplementary Material online.

The evaluation of the site-specific amino acid distributions based on the effective number of amino acids at every site showed, for both studied data (Fig. 6 and supplementary fig. S14, Supplementary Material online for test data and additional test data, respectively), that the empirical substitution models produced the least realistic site-specific amino acid variability, the MF model provided a more realistic fitting than the empirical model, and the SACS model produced the most realistic site-specific amino acid distributions and especially for sites related to the protein activity. For example, for sites 25, 50, and 84, which are directly related to the protein activity (Fig. 2), the SACS model produced amino acid variability very close to that observed in the real data (Fig. 6 and supplementary fig. S15, Supplementary Material online).

## Discussion

Most of currently available SCS models of protein evolution consider selection on the protein stability, and only a few additionally consider selection on the protein activity (i.e. Echave 2019) despite its fundamental role in nature (Saito et al. 1997; Bartlett et al. 2002; Echave et al. 2016; Jack et al. 2016; Echave 2021). Here, we present a substitution model of protein evolution with selection on both protein stability and activity, and we built it on the HIV-1 PR, which is a common HIV-1 molecular target for AIDS therapies (Debouck 1992). The model considers the enzyme–substrate binding-free energy, calculated by the MM-GBSA method and MD simulations (a methodology widely used to study the conformational changes that mutations can produce on complexes of HIV PR with drugs (Hou and Yu 2007; Jianzhong et al. 2020; Wang et al. 2020; Yu et al. 2022) despite requiring high computational costs [≍300 ns/day]), to produce site-specific substitution rates matrices and amino acid frequencies that can be used by probabilistic phylogenetic methods. The SACS model provided, in general, a better fitting with real data in comparison with the selected best-fitting empirical substitution model, the HIVpr empirical substitution model, and another relevant SCS model, particularly for protein regions involved in the enzymatic activity. Therefore, it suggested that the consideration of the enzyme–substrate binding affinity can provide useful information to model the evolution of functional protein regions.

### Fundaments and Development of the SACS Model

The SACS model showed that protein variants derived from amino acid substitutions at the protein core and flaps exhibit higher binding-free energy variations than those derived from substitution events in other regions of the protein. This is especially observed in variants with amino acids presenting physicochemical properties different to those of the WT (e.g. in substitutions from Ala to Arg, His, or Lys at site 28 in the protein core), which showed a lower affinity with the substrate and even decreased the protein folding stability (supplementary appendix, Supplementary Material online). We found that considering the HB occupancy in the substitution model was particularly informative to quantify the consequences of amino acid substitutions at sites of the protein core (supplementary fig. S5, Supplementary Material online), which could be expected since HBs are generally fundamental for the protein–substrate binding by displacing protein-bound water molecules into the bulk solvent (Salentin et al. 2014; Chen and Savidge 2015). The PR flaps affect the entry and accommodation of the substrate in the active site, modifying the structural conformation of the enzyme–substrate complex. Similarly to Yu et al. (2022), we considered the conformation of the flaps by the distance among the main PR catalytic sites and this distance also provided information about the consequences of substitution events at sites of the protein core and flaps (supplementary fig. S6, Supplementary Material online). Indeed, we included RG and SASA of each substituted amino acid backbone to study the modeling of the protein surface. Note that RG provides a measure of compactness, and thus, we expected similar predictions among stably folded complexes, as found for most of the sites (supplementary fig. S7, Supplementary Material online). SASA commonly provides information about amino acid conservation at both exposed and buried protein sites (Porto et al. 2005; Echave and Wilke 2017; Dubreuil and Levy 2021). Although Arenas et al. (2015) assumed regions with intermediate solvent accessibility as the most variable in the MF model (although this aspect was improved in posterior studies (Jimenez et al. 2018; Jiménez-Santos et al. 2018)), here, we additionally considered that a higher SASA can favor an increase of amino acid substitutions (Ramsey et al. 2011; Echave and Wilke 2017). For example, we identified that the WT amino acid at site 1 (Pro) produced an extremely low SASA score compared with other amino acid states, indicating that Pro is highly conserved at that site (supplementary fig. S8, Supplementary Material online). Altogether, while for protein core regions we obtained detailed information about selective constraints on the enzymatic activity (i.e. using the HB occupancy and catalytic box size), we barely found information about these selective constraints at the protein surface using RG and SASA. As a consequence, the SACS model is particularly useful to identify evolutionary constraints at functional protein regions, but further research (i.e. complementing with other models) could be required to improve the modeling of regions less involved in function.

### Phylogenetic Fitting of the SACS Model with Real Data of Variable Molecular Diversity

We compared the fitting (phylogenetic likelihood) of the SACS model, MF SCS model, best-fitting empirical substitution model selected with *ModelTest-NG* (HIVb), and HIVpr empirical substitution model, with several real data presenting variable molecular diversity, as well as with 3 additional real data. We performed this evaluation ignoring and considering in the models the variation of the global substitution rate among sites according to a gamma distribution that is traditionally used in phylogenetics. We found that the SACS model best fit with the data of high or intermediate levels of sequence identity (Fig. 3), either ignoring or considering variation of the global substitution rate among sites. However, and although the SACS model also best fit with most of the protein sites of data with any level of sequence identity (supplementary table S2, Supplementary Material online), this model was not globally preferred when fitting data with low sequence identity (Fig. 3). In this concern, the irregular performance of SCS models with study data of high molecular diversity is well known. Bordner and Mittelmann (2014) evaluated the fitting of several SCS models with 4 data sets of high sequence identity to find that those models outperform empirical substitution models but did not explore data with low sequence similarity. A similar finding was obtained with the MF SCS model (Arenas et al. 2015) although, in that study, the MF model was also evaluated with data presenting low sequence similarity showing in those cases a poor fitting in comparison with the best-fitting empirical substitution model. The reason for this is that most of the current SCS models (including MF) assume only one protein structure representative of the analyzed protein sequences. Thus, if the evaluated data include distant protein sequences (overall displaying a low sequence identity), those sequences far from the representative protein structure could be poorly modeled. We believe that this aspect also affected the SACS model at sites that are not related to the protein function and, in general, shows the general need of SCS models to consider more than one protein structure for modeling the evolution of data with highly divergent sequences. Despite this limitation, here, we show that considering selection on the protein activity (which is usually ignored in SCS models) provides a relevant improvement to model the evolution of most of the protein sites, especially those located in regions related to the protein activity (Fig. 5 and supplementary fig. S12 and table S2, Supplementary Material online). Indeed, the SACS model produced much higher likelihood estimates in the catalytic sites (from 25 to 27) than those obtained with the other evaluated substitution models and when fitting data with any level of sequence diversity. In addition, among the evaluated substitution models, the SACS model best-fitted the additional test data at both global (Fig. 4 and supplementary fig. S11, Supplementary Material online) and site-specific (supplementary table S3, Supplementary Material online) levels. These results also support the benefits from considering constraints on the protein activity in the development of substitution models.

Finally, we evaluated the substitution models in terms of site-specific amino acid variability through the effective number of amino acids (Fig. 6 and supplementary fig. S14, Supplementary Material online). We found that the empirical models, which ignore site-specific variation of amino acid frequencies among sites, produced highly unrealistic amino acid variability at any site. Next, the MF model, where the amino acid distribution can vary among sites due to constraints on the protein folding stability, was more realistic than the empirical model. Concerning the SACS model, which considers constraints on both stability and activity, it produced the most realistic amino acid variability and especially for sites related to the protein activity. The amino acid distributions produced by the SACS model were more realistic at sites directly related to the protein activity (i.e. 25, 50, and 84). These findings support that the SACS model, and in extension probably models with constraints on the protein activity, is particularly useful to more accurately predict protein evolution.

Although it is well known that protein evolution is driven by constraints not only on the protein folding stability but also on the protein activity (Bloom et al. 2004), most of the SCS models focused only on the stability probably due to the complexity of quantifying site-specific influences of substitution events on protein activity. Here, we formally investigated the benefits from accounting for the molecular activity, based on the binding affinity of the enzyme with its natural substrate, on the realism of the modeling of protein evolution. We identified constraints on the protein activity that can inform substitution models of evolution to improve the fitting with query data. A more realistic modeling of protein evolution is advantageous for traditional phylogenetics analyses (Lemmon and Moriarty 2004; Del Amparo et al. 2023; Del Amparo and Arenas 2023), but also the inclusion of the protein activity in the modeling has diverse potential applications such as a likely improvement of the prediction of substitution events related to the activity in protein drug targets of pathogens (Arenas and Posada 2010; Guerin et al. 2022; Patel et al. 2022) or the reconstruction of ancestral proteins with realistic activity in paleoenzymology (Thomson et al. 2005; Kothe et al. 2006; Yamashiro et al. 2010; Perez-Jimenez et al. 2011). We believe that the inclusion of the protein activity into SCS models of protein evolution that we present can still be improved by adding selection on other molecular processes (i.e. long-distance epistatic interactions and protein–protein interactions), as well as additional parameterization of the protein function as that presented by Echave (2019), and thus, further research in this direction would be convenient.

## Supplementary Material

Supplementary material is available at *Molecular Biology and Evolution* online.

## Supplementary Material

msae026_Supplementary_Data

## Data Availability

The data for building the SACS model, the derived site-specific substitution rates matrices, and amino frequencies at the equilibrium and the test data to evaluate the substitution models are available from Zenodo repository at the URL https://doi.org/10.5281/zenodo.10559982.
