# Supplementary material for: Substitution Models of Protein Evolution with Selection on Enzymatic Activity
Source: Mol Biol Evol. 2024 Feb 5;41(2):msae026. doi: 10.1093/molbev/msae026 (PMC10873502; doi:10.1093/molbev/msae026)

Supplementary Material

Substitution models of protein evolution with selection on enzymatic activity

The supplementary material includes Tables S1-S3 and Figures S1-S15.

Supplementary tables

Table S1. Predicted protein-substrate binding-free energy of the wild-type structure through molecular dynamics simulations with variable simulation time and trajectory sampled frames. Protein-substrate binding-free energies (ΔG, kcal/mol) obtained for the wild-type enzyme with its substrate using molecular dynamics (MD) simulations with variable simulation time (1, 5, 10, 20, and 50 ns) and trajectory sampled frames (50, 100, 200, 500, and 1000 frames). We performed three independent runs (replicates) that are presented in every table.

	Number of frames / Simulation time (ns)	50	100	200	500	1000
Replicate 1	1	-86.8049	-86.8423	-87.0427	-87.237	-87.2419
	5	-86.1664	-85.4623	-84.7345	-84.8175	-84.8221
	10	-87.3463	-87.3787	-87.0021	-87.0073	-86.9444
	20	-90.4164	-91.5081	-91.2269	-90.8565	-90.7786
	50	-94.963	-94.4612	-94.3247	-94.6312	-94.3003

	Number of frames / Simulation time (ns)	50	100	200	500	1000
Replicate 2	1	-93.2818	-93.3103	-93.9183	-93.9345	-93.9902
	5	-94.9546	-95.3087	-95.4115	-95.3459	-95.4203
	10	-93.8079	-93.5384	-93.289	-93.6863	-93.6351
	20	-94.271	-94.3937	-94.1261	-94.33	-94.0771
	50	-96.0919	-96.037	-95.6295	-96.4671	-96.3282

	Number of frames / Simulation time (ns)	50	100	200	500	1000
Replicate 3	1	-88.4657	-89.2501	-89.0839	-89.0233	-88.7751
	5	-90.8706	-90.3229	-90.7996	-90.6642	-90.7403
	10	-91.742	-92.2644	-92.092	-92.1935	-92.0804
	20	-94.5183	-94.5012	-94.5829	-94.402	-94.5899
	50	-97.0635	-96.7968	-96.5834	-97.1055	-96.8875

Table S2. Site-specific fitting of every substitution model with test data of different molecular diversity. Number of HIV PR sites where the SACS, HIVpr, HIVb or MF models provided a better fitting (BIC score) with the observed data compared to the other substitution models when ignoring (table above) or considering (table below) substitution rate variation among sites according to a Gamma distribution (+G). Every row refers to a test dataset with particular sequence identity (second column). For every of the three levels of studied sequence identity, a subsequent row indicates the percentage of sites that were better fitted with every substitution model.

**Data**	**Sequence identity**	**Number of sites that best-fitted with a substitution model**			
		**SACS**	**HIVpr**	**HIVb**	**MF**
Data 1	0.933	75	9	3	12
Data 2	0.933	76	8	1	14
Data 3	0.934	78	9	3	9
		**77.10%**	**8.75%**	**2.36%**	**11.78%**
Data 1	0.860	54	22	9	14
Data 2	0.867	56	24	6	13
Data 3	0.864	47	30	9	13
		**52.86%**	**25.59%**	**8.08%**	**13.47%**
Data 1	0.787	33	33	13	20
Data 2	0.783	36	36	11	26
Data 3	0.781	40	29	8	22
		**36.70%**	**33.00%**	**10.77%**	**19.53%**

**Data**	**Sequence identity**	**Number of sites that best-fitted with a substitution model**			
		**SACS +G**	**HIVpr +G**	**HIVb +G**	**MF +G**
Data 1	0.933	80	6	4	9
Data 2	0.933	81	6	1	11
Data 3	0.934	83	7	3	6
		**82.15%**	**6.40%**	**2.69%**	**8.75%**
Data 1	0.860	60	17	7	15
Data 2	0.867	67	16	5	11
Data 3	0.864	65	17	5	12
		**64.65%**	**16.84%**	**5.72%**	**12.79%**
Data 1	0.787	46	24	10	19
Data 2	0.783	45	30	11	13
Data 3	0.781	49	24	13	13
		**47.14%**	**26.26%**	**11.45%**	**15.15%**

Table S3. Site-specific fitting of every substitution model with the additional test data. Number of HIV PR sites where the SACS, HIVpr, HIVb or MF models provided a better fitting (BIC score) with the observed data compared to the other substitution models when ignoring (table above) or considering (table below) substitution rate variation among sites according to a Gamma distribution (+G). Every row refers to an additional test dataset (see main text). For every dataset, a subsequent row indicates the percentage of sites that were better fitted with every substitution model.

**Data**	**Number of sites that best-fitted with a substitution model**			
	**SACS**	**HIVpr**	**HIVb**	**MF**
Additional data 1	77	13	5	4
Additional data 2	61	20	9	9
Additional data 3	63	17	9	10
	**67.68%**	**16.84%**	**7.74%**	**7.74%**

**Data**	**Number of sites that best-fitted with a substitution model**			
	**SACS +G**	**HIVpr +G**	**HIVb +G**	**MF +G**
Additional data 1	81	8	5	5
Additional data 2	58	20	13	8
Additional data 3	66	16	7	10
	**69.02%**	**14.81%**	**8.42%**	**7.74%**

Supplementary figures

Figure S1. Predicted P factor for PR variants with each possible amino acid in three illustrative sites located at the protein core and surface. Factor *P* (see main text) predicted for PR variants with every possible amino acid at representative sites of the protein core (sites 8 and 29) and protein surface (site 18). The protein variant with the WT amino acid is shown in light gray. The plots show the sum of normalized values of the four independent parameters (see Methods) and error bars indicate its mean absolute deviation (MAD).

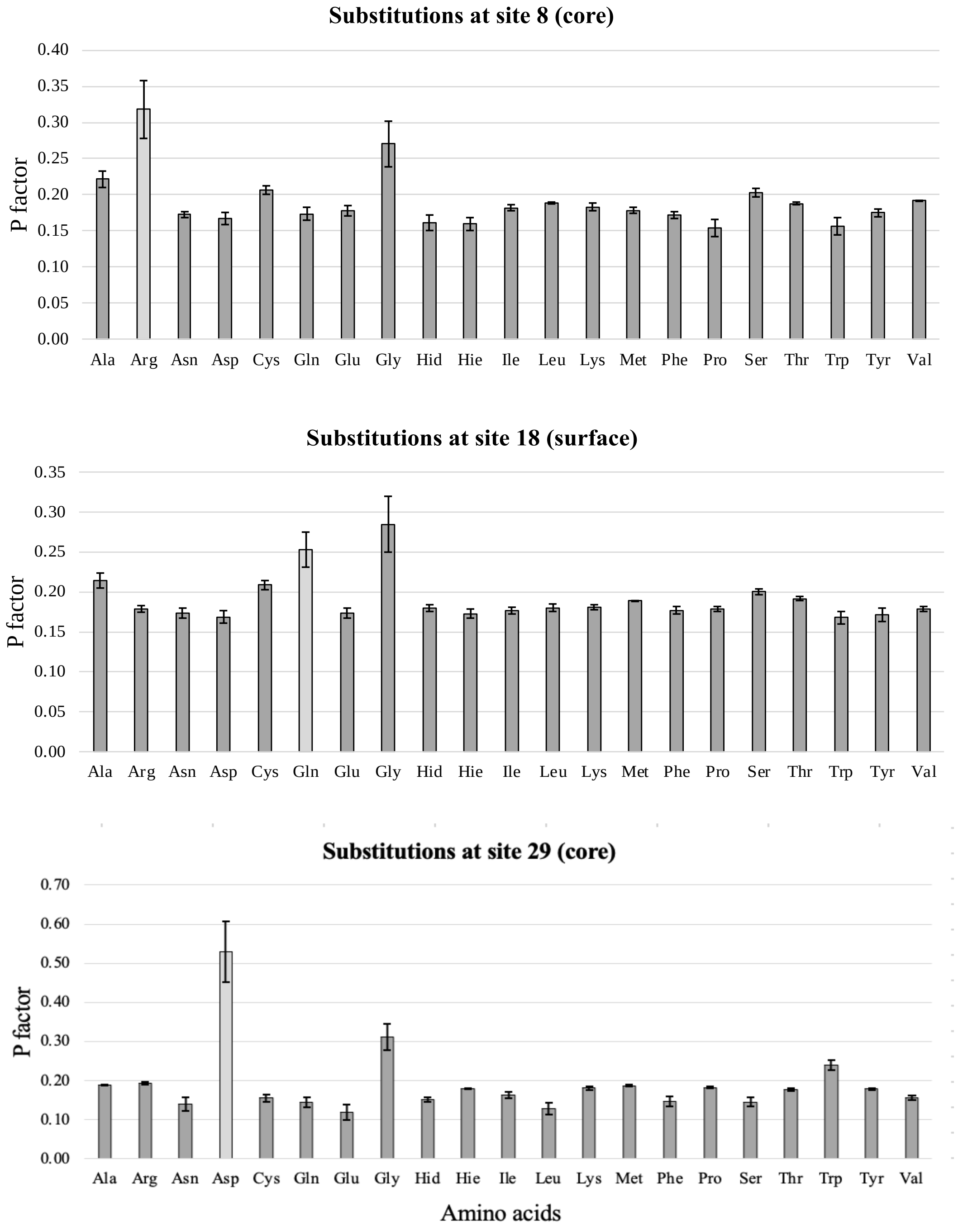

Figure S2. Predicted binding free energy for PR variants with each possible amino acid in three illustrative sites located at the protein core and surface. The figure shows the predicted binding free energy in PR variants with every possible amino acid at representative sites of the protein core (sites 8 and 29) and protein surface (site 18). The protein variant with the WT amino acid is shown in light gray. The figure shows the mean of three independent predictions (see Methods) and error bars indicate the 95% confidence interval from the mean.

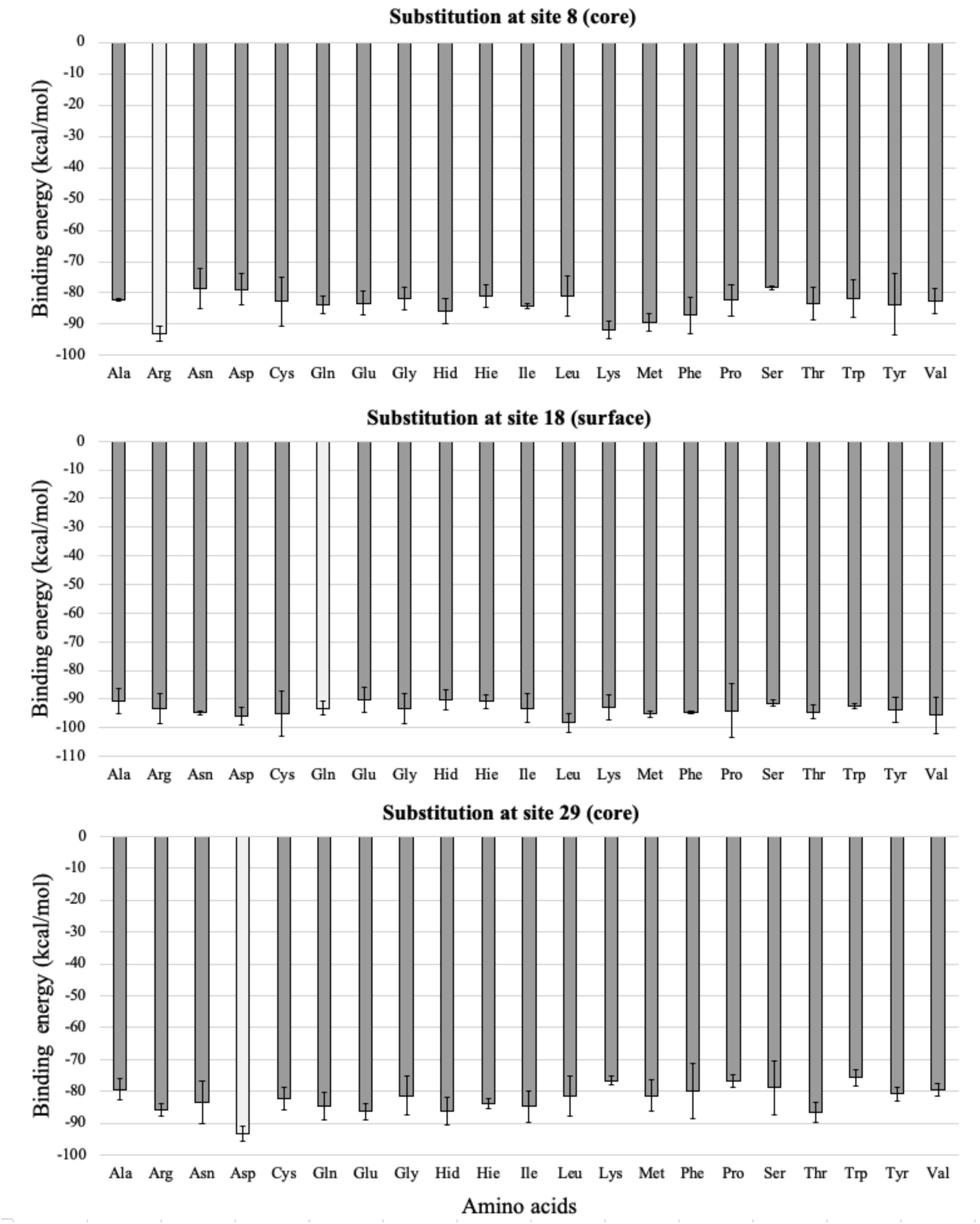

Figure S3. Variation of the binding free energy of the HIV-1 PR with its natural substrate among all the protein variants with every amino acid at every protein site. Standard deviation of the predicted binding free energy in all the protein variants with every amino acid at every protein site. A high standard deviation at a site means that applying amino acid substitutions at that site can affect the binding free energy of the protein with the substrate. These results are depicted on the protein structure in Figure 2.

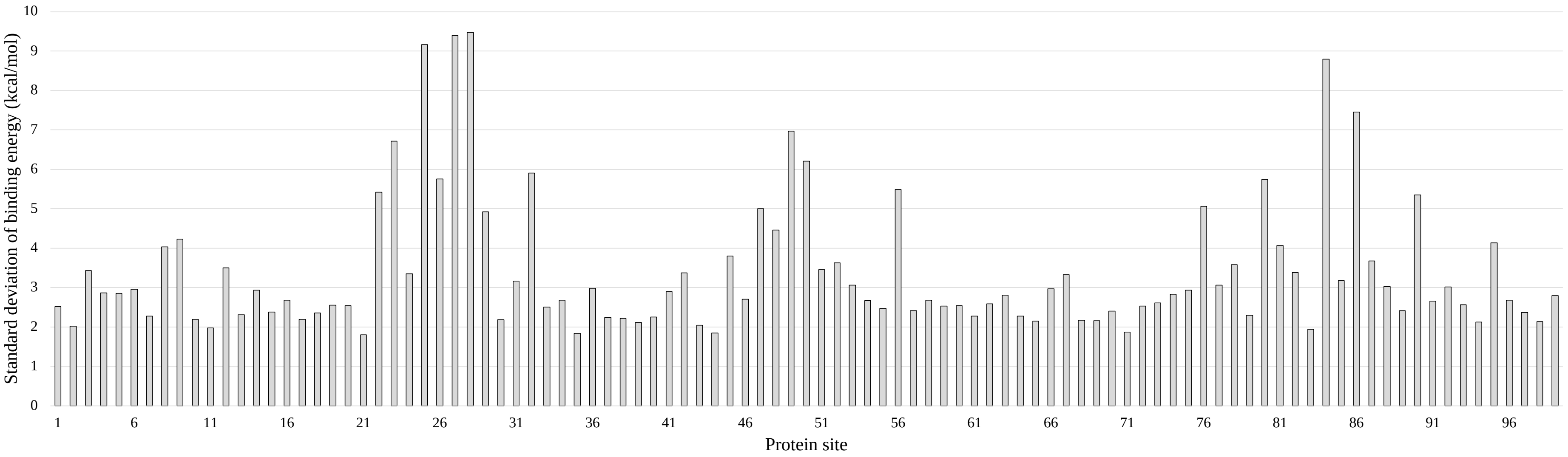

Figure S4. Mean of the root mean square deviation among all the variants of the HIV-1 PR with its natural substrate where each variant has every amino acid at every protein site. The plot shows the mean of the root mean square deviation (RMSD) of the protein backbone of variants with all the possible amino acids at every protein site. The results are derived from a 20 ns MD simulation using 1000 trajectory sampled frames. Error bars correspond to the standard error of the mean. This plot provides a checking of the methods (see main text), where a site with high RMSD can suggest a possible source of error. We did not find major RMSD differences among sites.

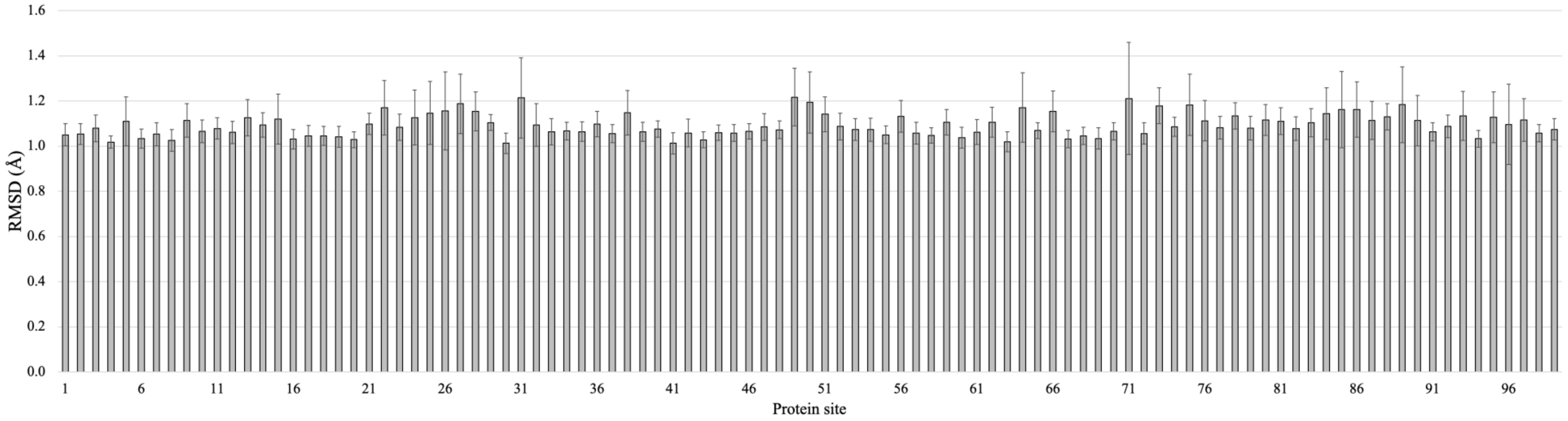

Figure S5. Standard deviation of the protein substrate hydrogen bond (HB) occupancy of the HIV-1 PR with its natural substrate among all the protein variants with every amino acid at every protein site. The figure shows the standard deviation of the protein HB occupancy among the variants derived from all the possible amino acids at every site. The results are based on a 20 ns MD simulation using 1000 trajectory sampled frames. HB occupancy was calculated as the sum of the number of frames where every protein amino acid has HB with the substrate. Note that a high standard deviation of HB occupancy at a site means that applying amino acid substitutions at that site can affect the HB occupancy (the amino acid change can affect the enzyme-substrate binding).

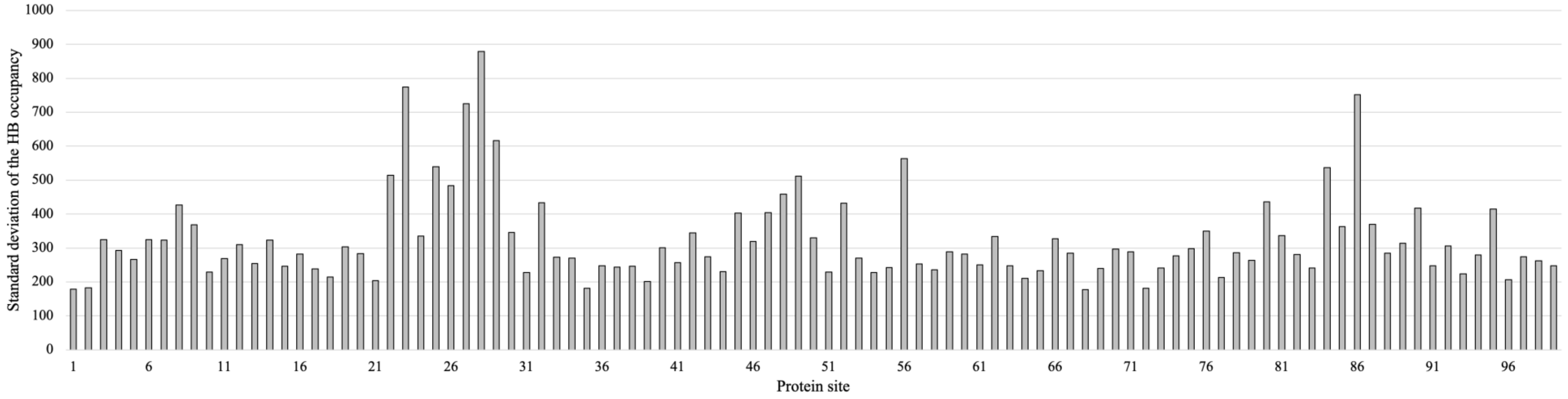

Figure S6. Standard deviation of the catalytic box size of the HIV-1 PR with its natural substrate among all the protein variants with every amino acid at every protein site. The figure shows the standard deviation of the protein catalytic box size among the variants derived from all the possible amino acids at every site. The results are based on a 20 ns MD simulation using 1000 trajectory sampled frames. The catalytic box size is calculated as the sum of the distance (Å) between the backbone of catalytic sites (25, 25', 50 and 50'). Note that a high standard deviation of the catalytic box size at a site means that applying amino acid substitutions at that site can affect the catalytic box size (the amino acid change can affect the enzyme-substrate binding).

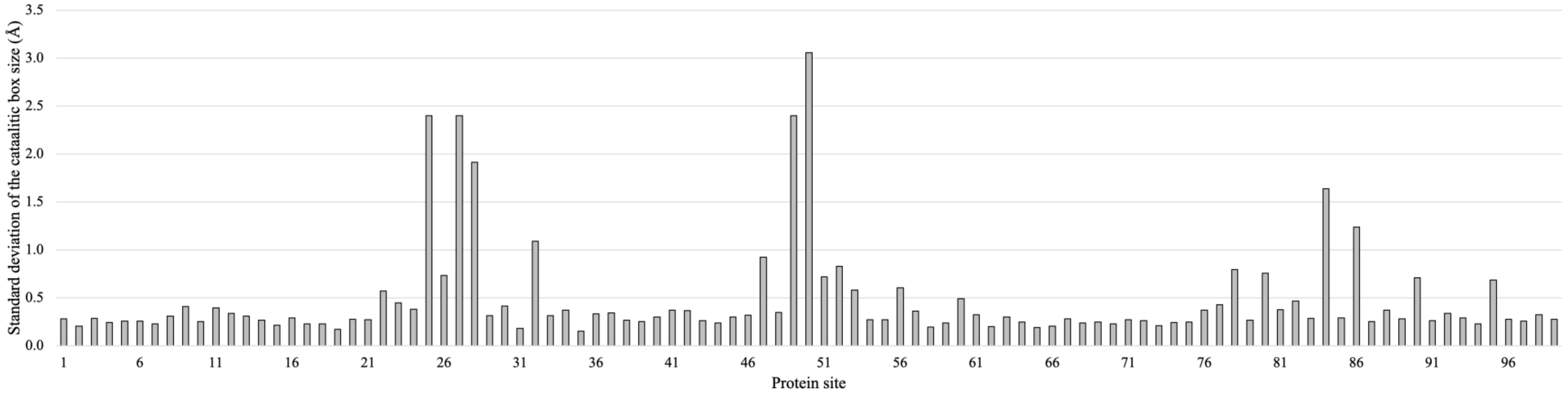

Figure S7. Standard deviation of the radius of gyration (RG) of the HIV-1 PR with its natural substrate among all the protein variants with every amino acid at every protein site. The figure shows the standard deviation of the protein backbone RG among the variants derived from all the possible amino acids at every site. The results are based on a 20 ns MD simulation using 1000 trajectory sampled frames. Note that a high standard deviation of the radius of gyration at a site means that applying amino acid substitutions at that site can affect the radius of gyration (the amino acid change can affect the enzyme-substrate binding).

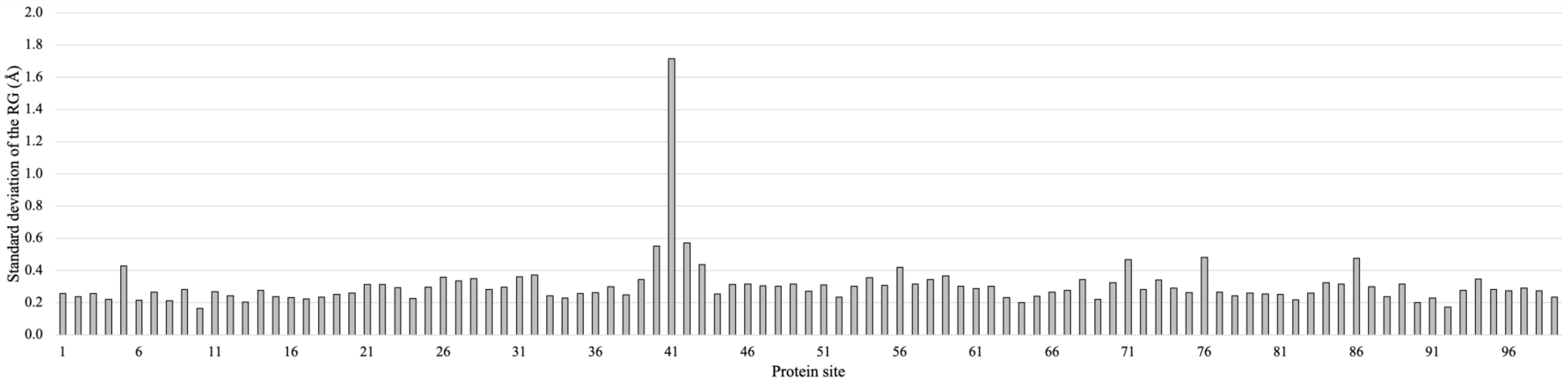

Figure S8. Standard deviation of the solvent-accessible surface area (SASA) of the HIV-1 PR with its natural substrate among all the protein variants with every amino acid at every protein site. The figure shows the standard deviation of the solvent-accessible surface area (SASA) among the variants derived from all the possible amino acids at every site. The results are based on a 20 ns MD simulation using 1000 trajectory sampled frames. Note that a high standard deviation of SASA at a site means that applying amino acid substitutions at that site dramatically affects SASA (the amino acid change can affect the enzyme-substrate stability and binding).

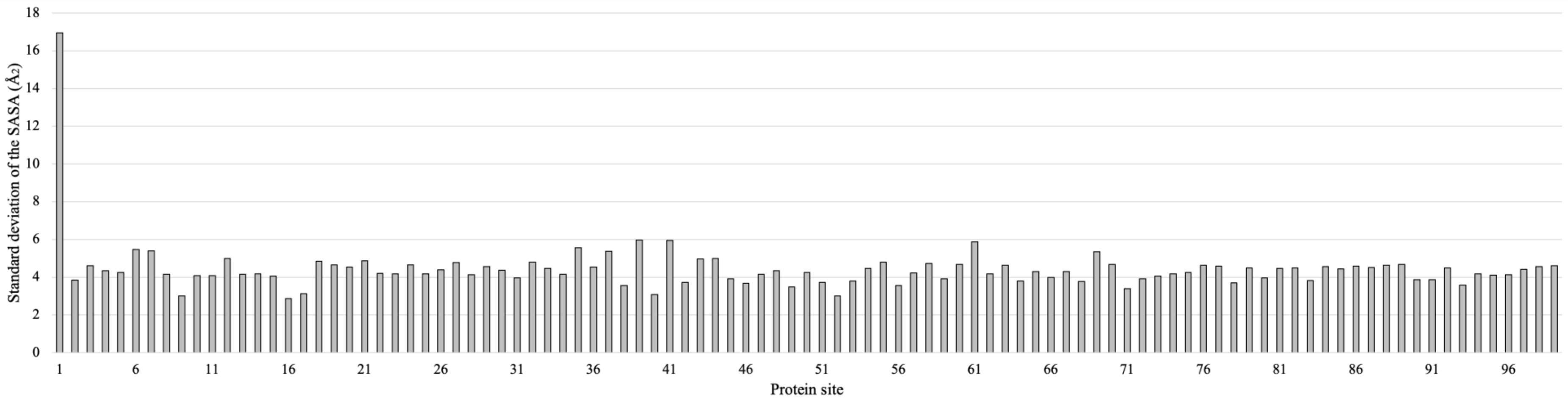

Figure S9. Fitting of the different substitution models of protein evolution with test data of different molecular diversity through phylogenetic likelihood. The figure shows the Log-likelihood (A) and Akaike Information Criterion (AIC) scores (B) obtained for the structure and activity constrained substitution (SACS) model, the mean-field (MF) structurally constrained substitution model, the HIVpr empirical substitution model and the HIVb empirical substitution model (that was selected as the best-fitting empirical substitution model for the corresponding data among the empirical substitution models available in *ModelTest-NG*) for several data with different sequence identity levels (data with higher sequence identity are shown on the left and data with lower sequence identity are shown on the right). Note that a higher log-likelihood and a lower AIC score indicate a better fitting of that model with the data. The corresponding BIC scores are shown in Figure 3.

A

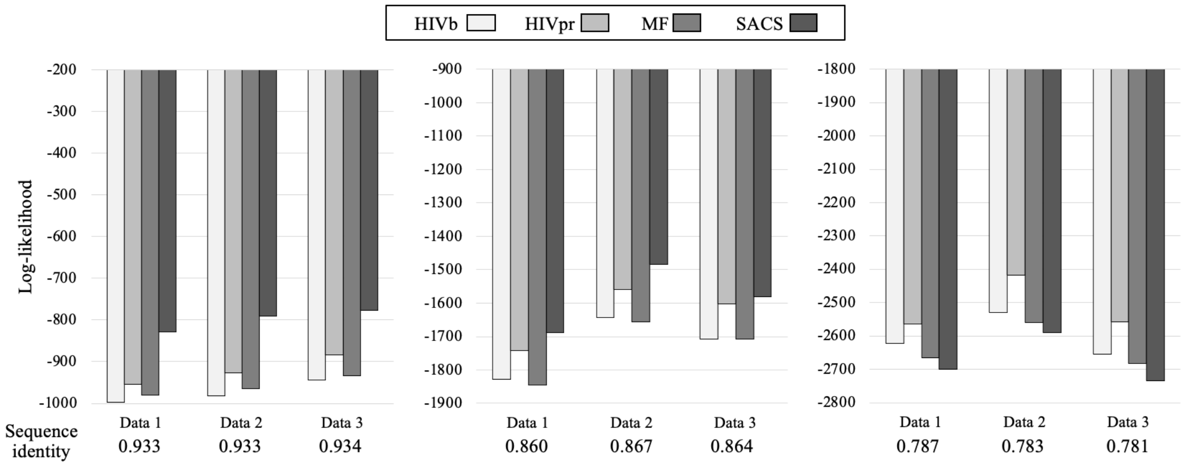

B

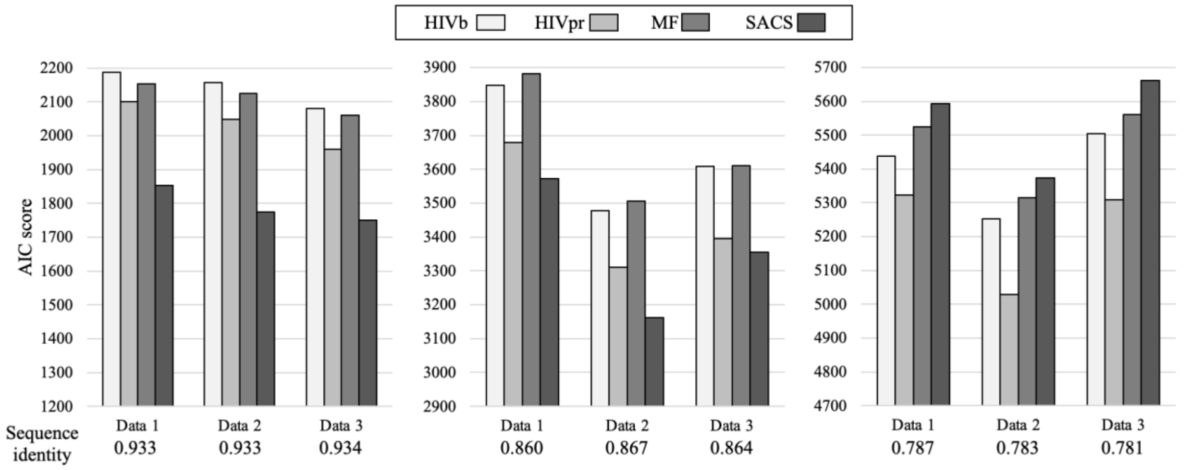

Figure S10. Fitting of the different substitution models accounting for substitution rate variation among sites according to a Gamma distribution with test data of different molecular diversity through phylogenetic likelihood. Log-likelihood (A), Bayesian Information Criterion (BIC) scores (B), and Akaike Information Criterion (AIC) scores (C) obtained for the structure and activity constrained substitution (SACS) model, the mean-field (MF) structurally constrained substitution model, the HIVpr empirical substitution model and the HIVb empirical substitution model (that was selected as the best-fitting empirical substitution model for the corresponding data among the empirical substitution models available in *ModelTest-NG*), where all these models considered variation of the global substitution rate among sites according to a Gamma distribution (+G), for fitting data with different sequence identity levels (data with higher sequence identity are shown on the left and data with lower sequence identity are shown on the right). Note that a higher (less negative) log-likelihood and a lower BIC and AIC scores indicate a better fitting of the model with the data.

A

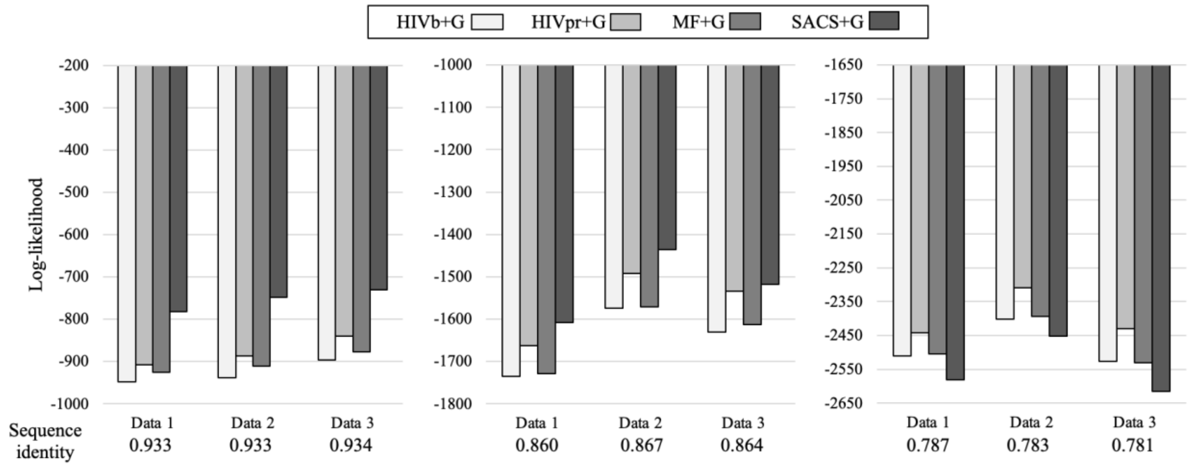

B

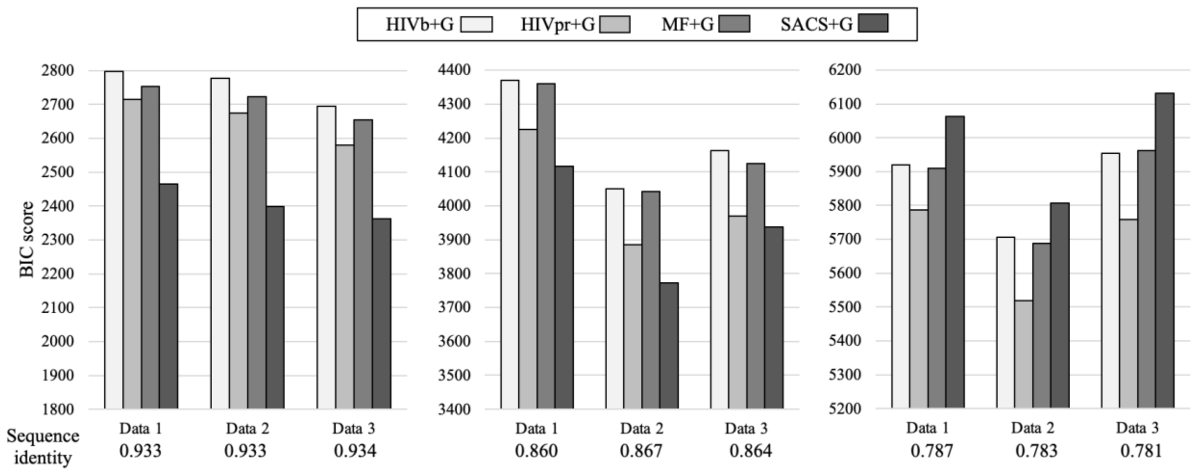
HIVb+G
HIVpr+G
MF+G
SACS+G
BIC score
Sequence identity
Data 1
0.933
Data 2
0.933
Data 3
0.934
Data 1
0.860
Data 2
0.867
Data 3
0.864
Data 1
0.787
Data 2
0.783
Data 3
0.781

C

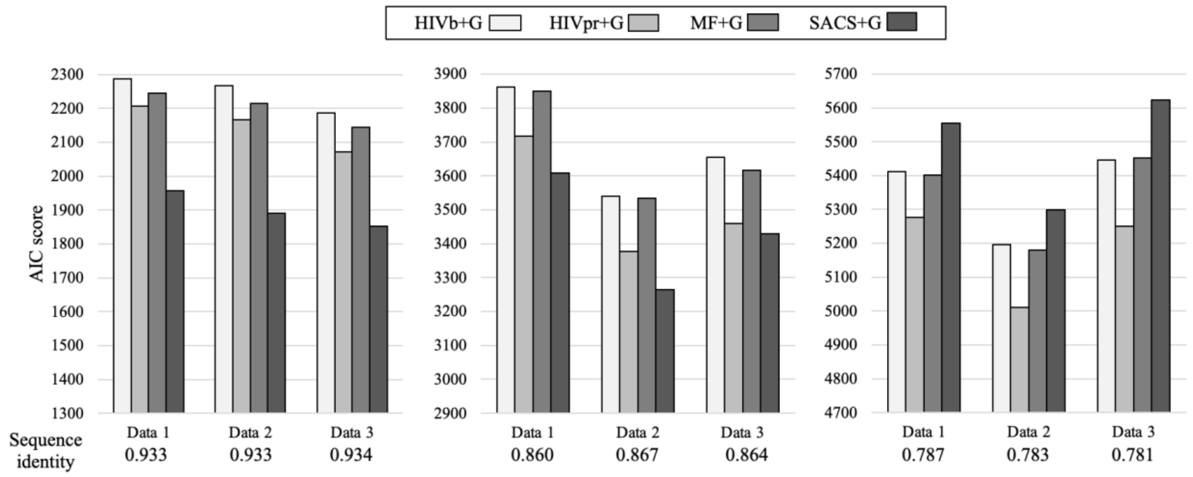
HIVb+G
HIVpr+G
MF+G
SACS+G
AIC score
Sequence identity
Data 1
0.933
Data 2
0.933
Data 3
0.934
Data 1
0.860
Data 2
0.867
Data 3
0.864
Data 1
0.787
Data 2
0.783
Data 3
0.781

Figure S11. Fitting of the different substitution models without and with global substitution rate variation among sites according to a Gamma distribution with the additional test data through phylogenetic likelihood. The figure shows the log-likelihood (first column), Bayesian Information Criterion (BIC) scores (second column) and Akaike Information Criterion (AIC) scores (third column) obtained for the activity constrained substitution (SACS) model, the mean-field (MF) structurally constrained substitution model, the HIVpr empirical substitution model and the HIVb empirical substitution model (that was selected as the best-fitting empirical substitution model for the corresponding data among the empirical substitution models available in *ModelTest-NG*), without (A) and with (B) the substitution models considering variation of the global substitution rate among sites according to a Gamma distribution (+G), for fitting the additional. The BIC scores for the substitution models without variation of the global substitution rate among sites are presented in Figure 4 (main text).

A

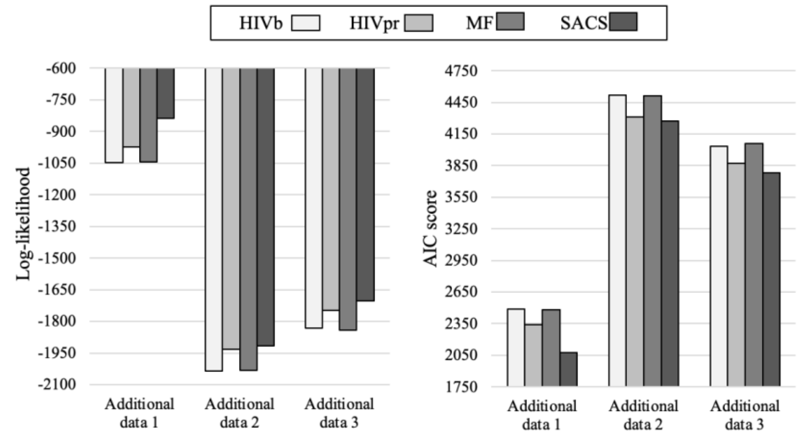

B

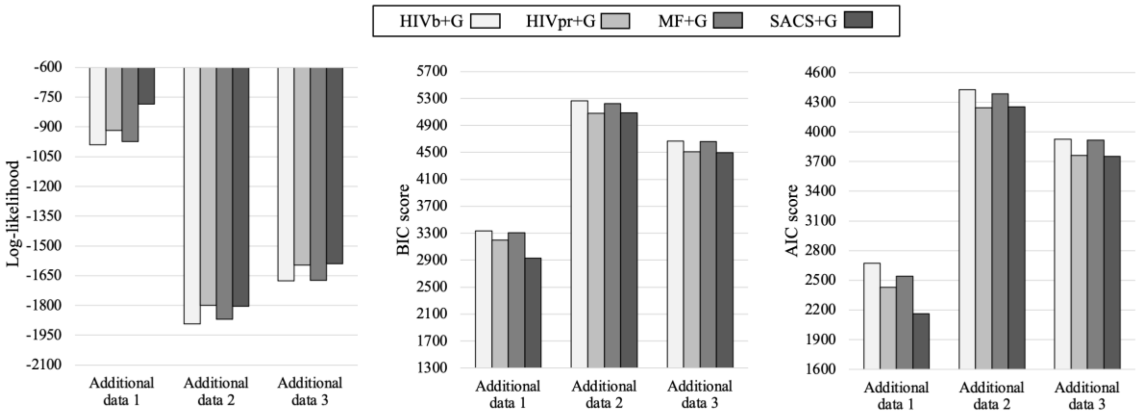

Figure S12. Comparison of the different substitution models by site-specific phylogenetic likelihood with data of variable molecular diversity. The plots show the difference of phylogenetic likelihood (lk) between the structure and activity constrained substitution (SACS) model, the HIVpr empirical substitution model and the HIVb empirical substitution model (that was selected as the best-fitting empirical substitution model for the corresponding data among the empirical substitution models available in *ModelTest-NG*) and, between the SACS model and the mean-field (MF) structurally constrained substitution model. The analyses were performed for the data with high sequence identity (0.933, 0.933 and 0.934), intermediate sequence identity (0.860, 0.867 and 0.864) and low sequence identity (0.787, 0.783 and 0.781), shown in rows A, B and C, respectively. Note that positive Δlk indicate a better fitting of the SACS model with the data.

A

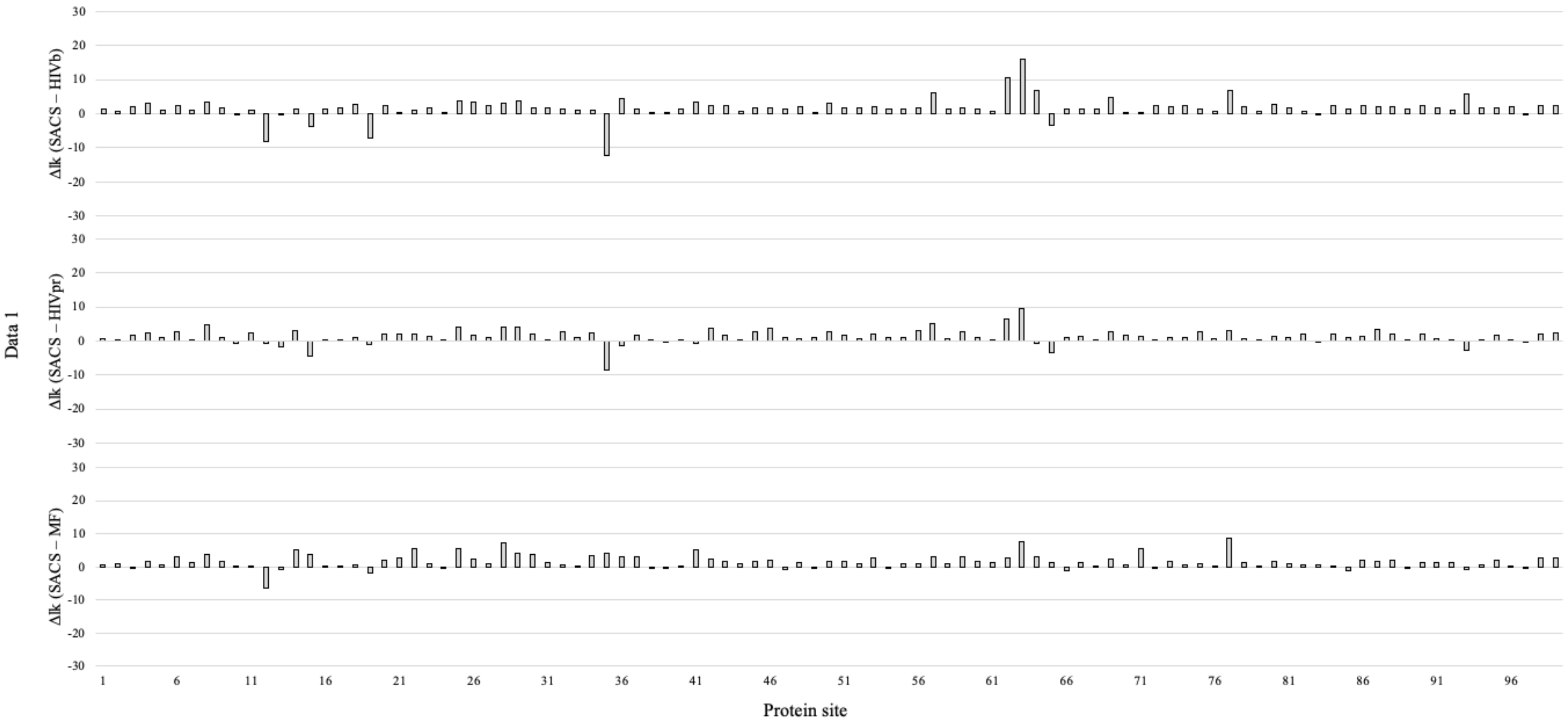

Data 1
Δlk (SACS – HIVb)
Δlk (SACS – HIVpr)
Δlk (SACS – MF)
30
20
10
0
-10
-20
-30
1
6
11
16
21
26
31
36
41
46
51
56
61
66
71
76
81
86
91
96
Protein site

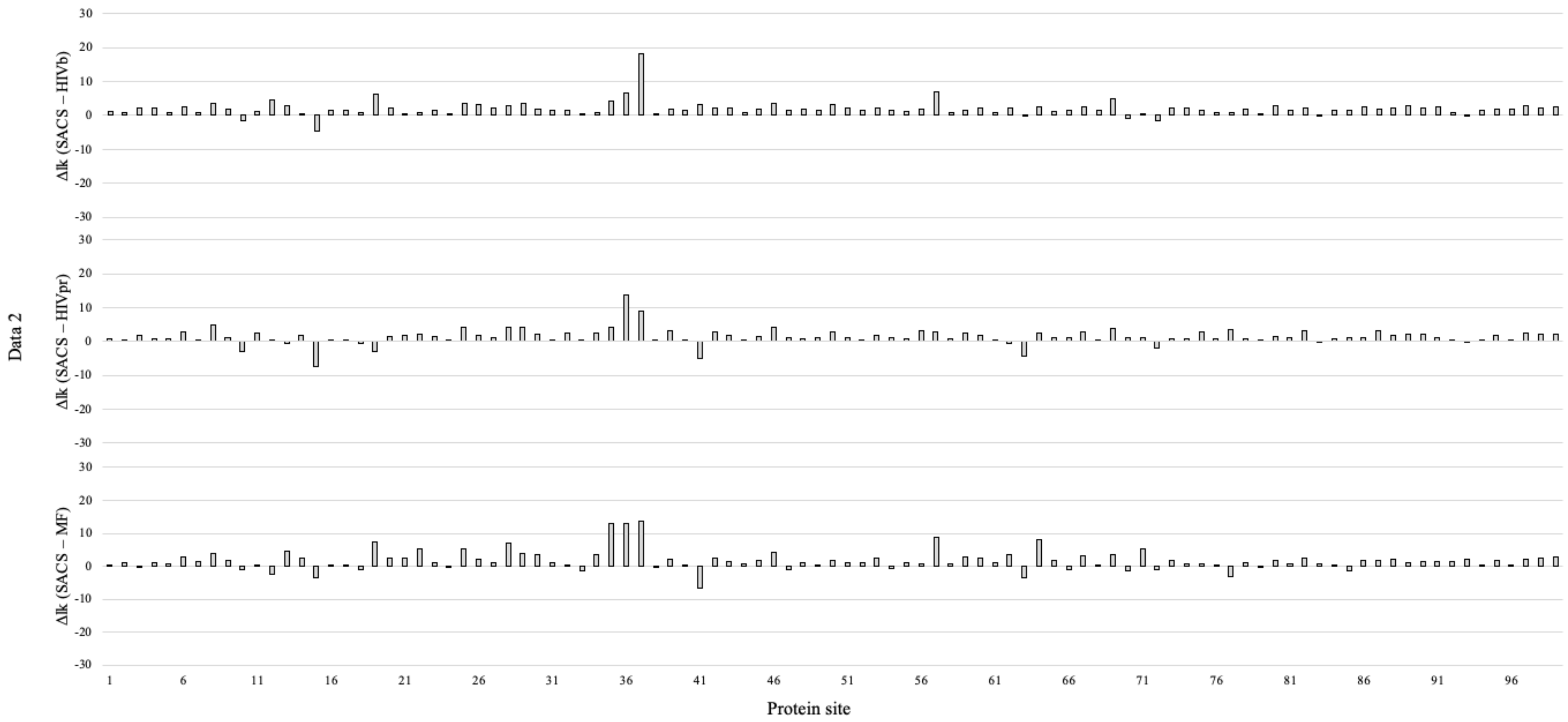
Data 2
Δlk (SACS – HIVb)
Δlk (SACS – HIVpr)
Δlk (SACS – MF)
30
20
10
0
-10
-20
-30
1
6
11
16
21
26
31
36
41
46
51
56
61
66
71
76
81
86
91
96
Protein site

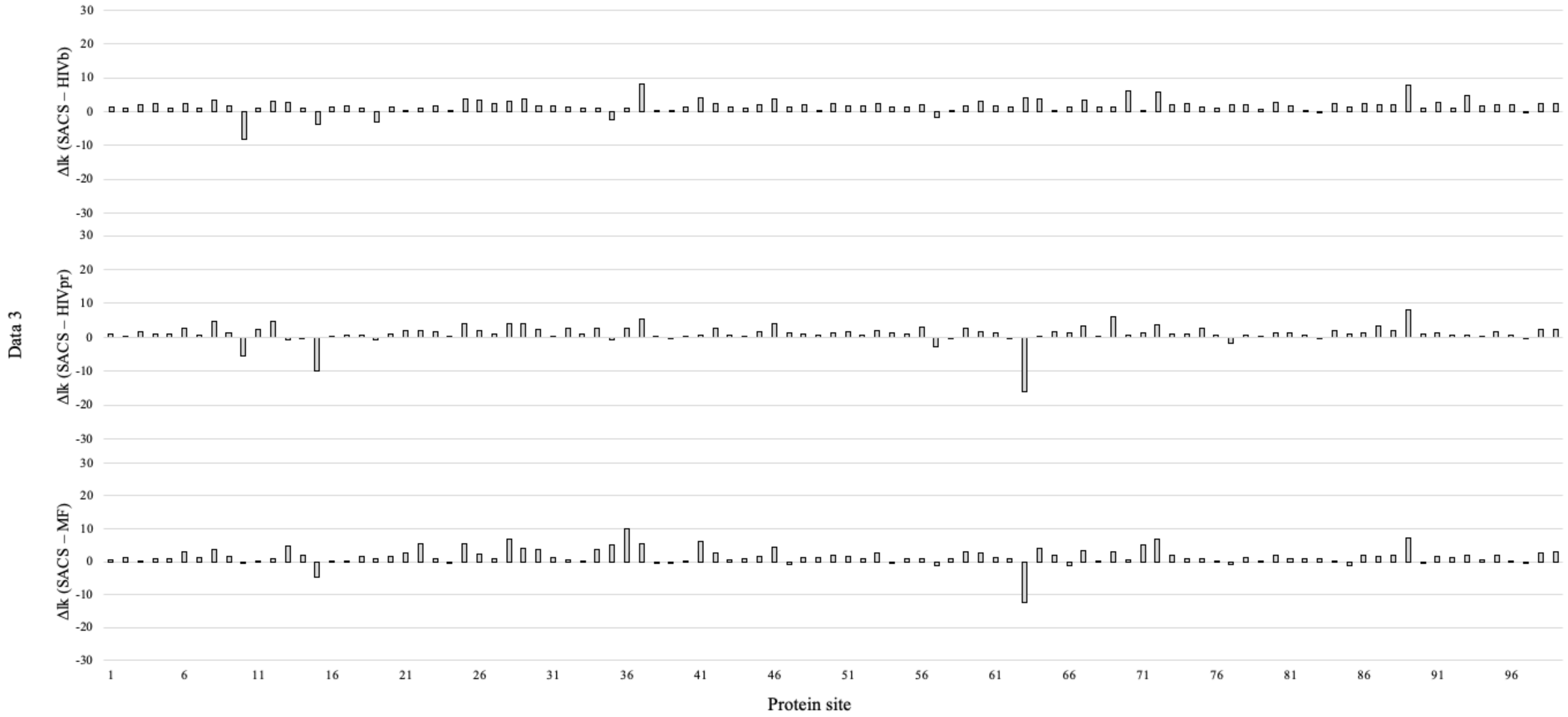

Data 3
Δlk (SACS – HIVb)
Δlk (SACS – HIVpr)
Δlk (SACS – MF)
30
20
10
0
-10
-20
-30
1
6
11
16
21
26
31
36
41
46
51
56
61
66
71
76
81
86
91
96
Protein site

B

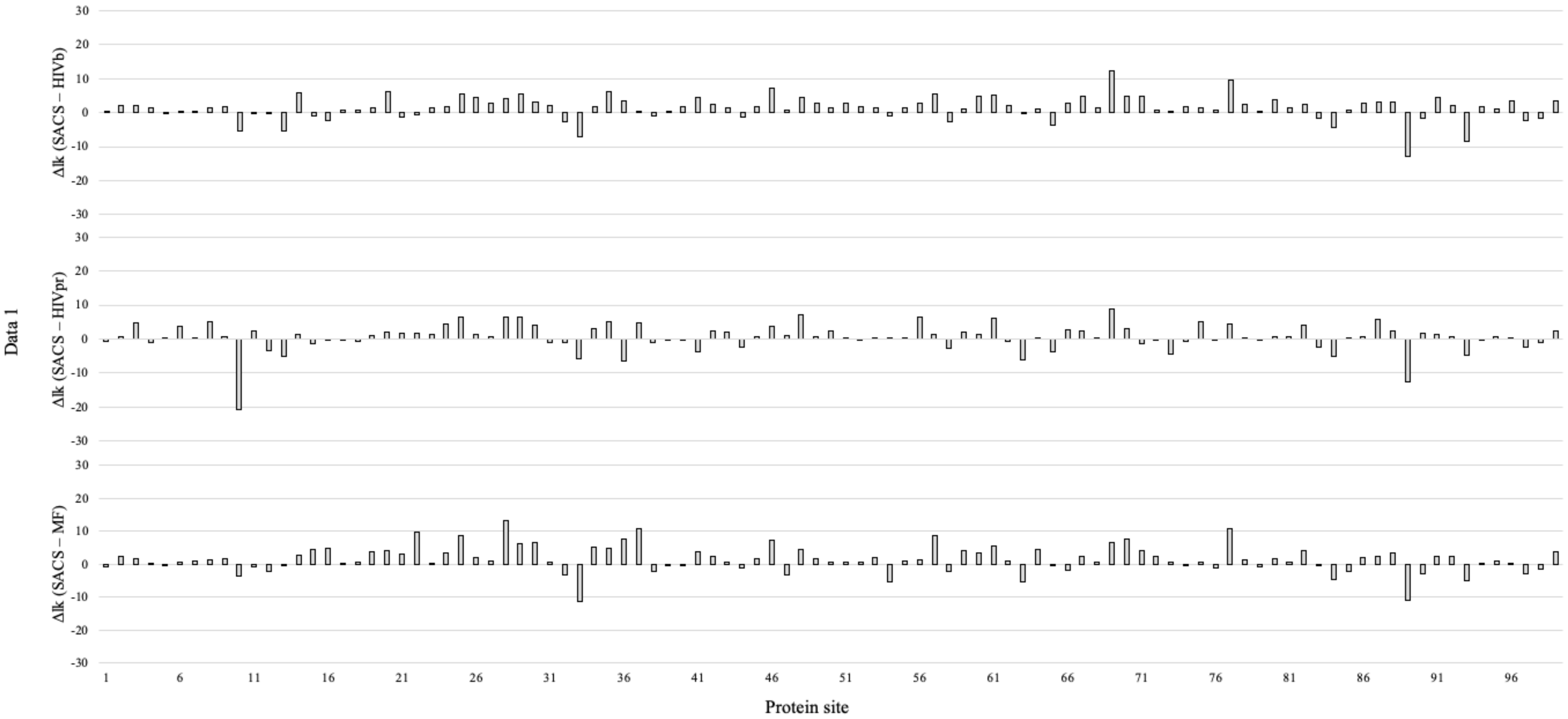

Data 1
Δlk (SACS – HIVb)
Δlk (SACS – HIVpr)
Δlk (SACS – MF)
30
20
10
0
-10
-20
-30
1
6
11
16
21
26
31
36
41
46
51
56
61
66
71
76
81
86
91
96
Protein site

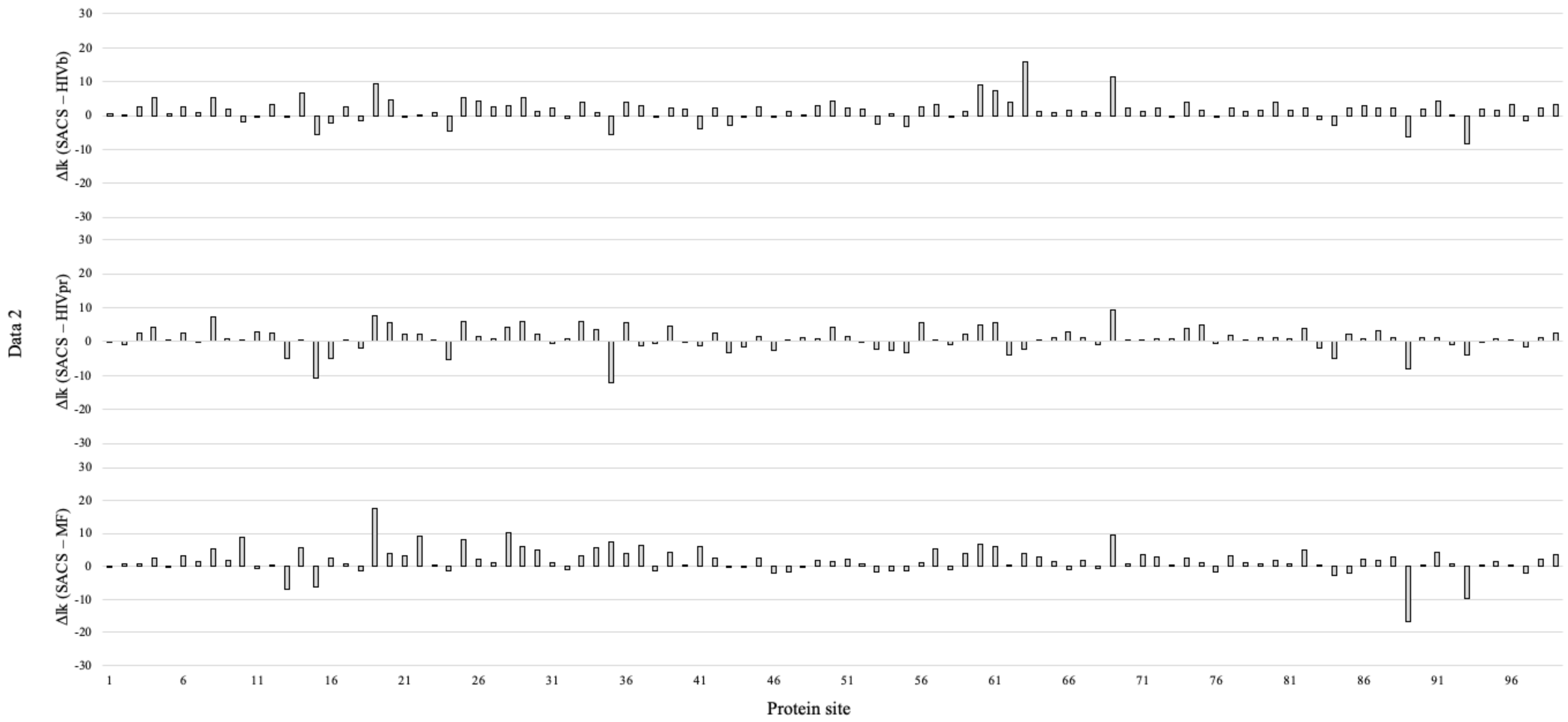
Data 2
Δlk (SACS – HIVb)
Δlk (SACS – HIVpr)
Δlk (SACS – MF)
30
20
10
0
-10
-20
-30
1
6
11
16
21
26
31
36
41
46
51
56
61
66
71
76
81
86
91
96
Protein site

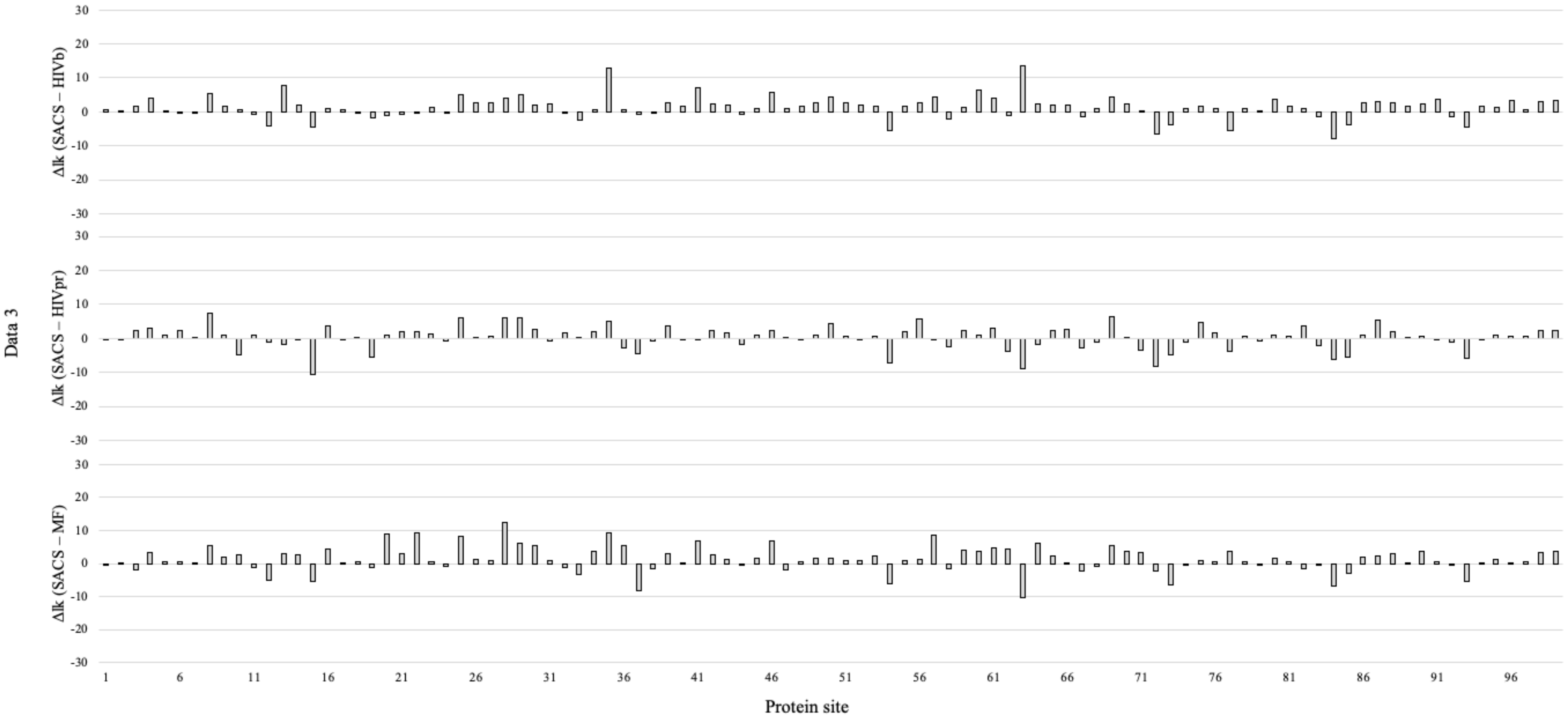

Data 3
Δlk (SACS – HIVb)
Δlk (SACS – HIVpr)
Δlk (SACS – MF)
30
20
10
0
-10
-20
-30
1
6
11
16
21
26
31
36
41
46
51
56
61
66
71
76
81
86
91
96
Protein site

C

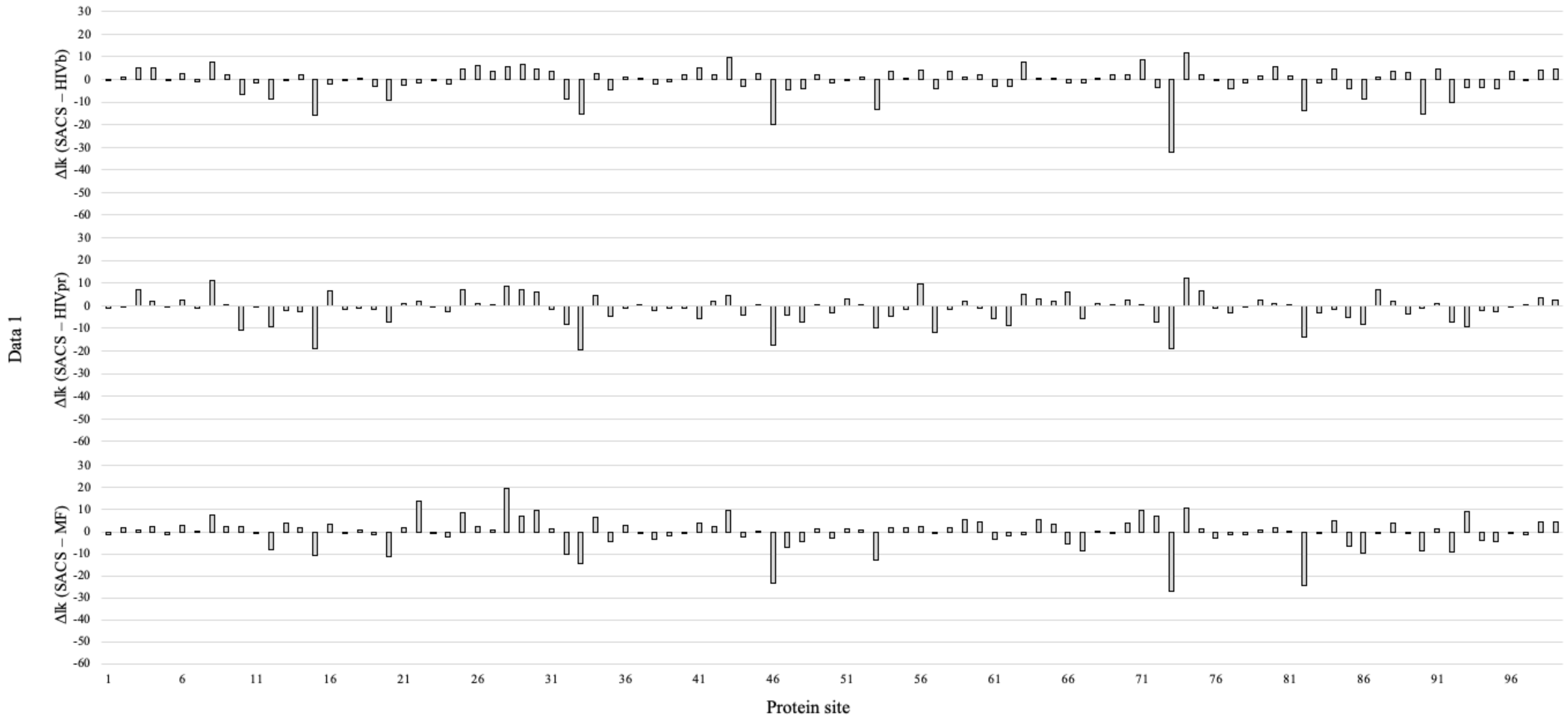
Data 1
Δlk (SACS – HIVb)
Δlk (SACS – HIVpr)
Δlk (SACS – MF)
30
20
10
0
-10
-20
-30
-40
-50
-60
1
6
11
16
21
26
31
36
41
46
51
56
61
66
71
76
81
86
91
96
Protein site

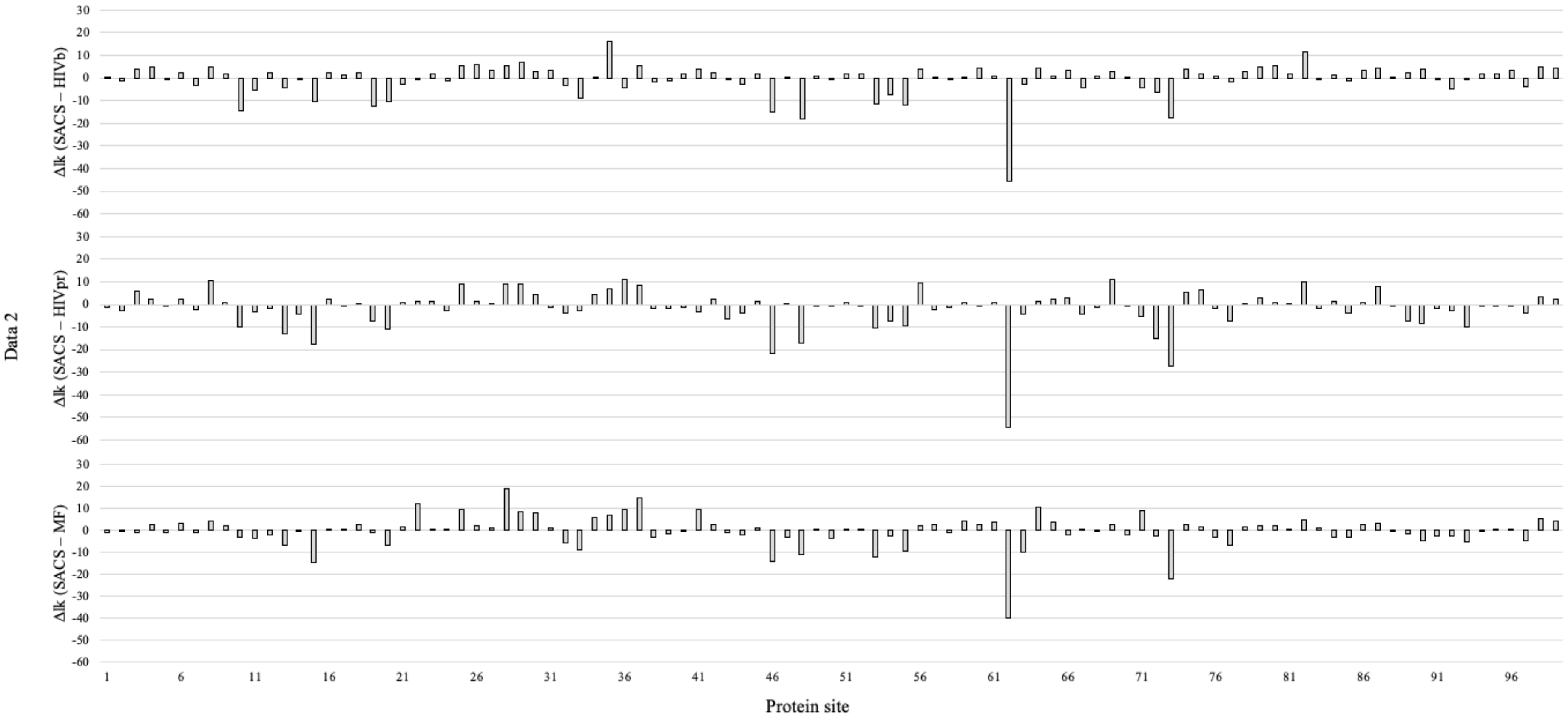
Data 2
Δlk (SACS – HIVb)
Δlk (SACS – HIVpr)
Δlk (SACS – MF)
30
20
10
0
-10
-20
-30
-40
-50
-60
1
6
11
16
21
26
31
36
41
46
51
56
61
66
71
76
81
86
91
96
Protein site

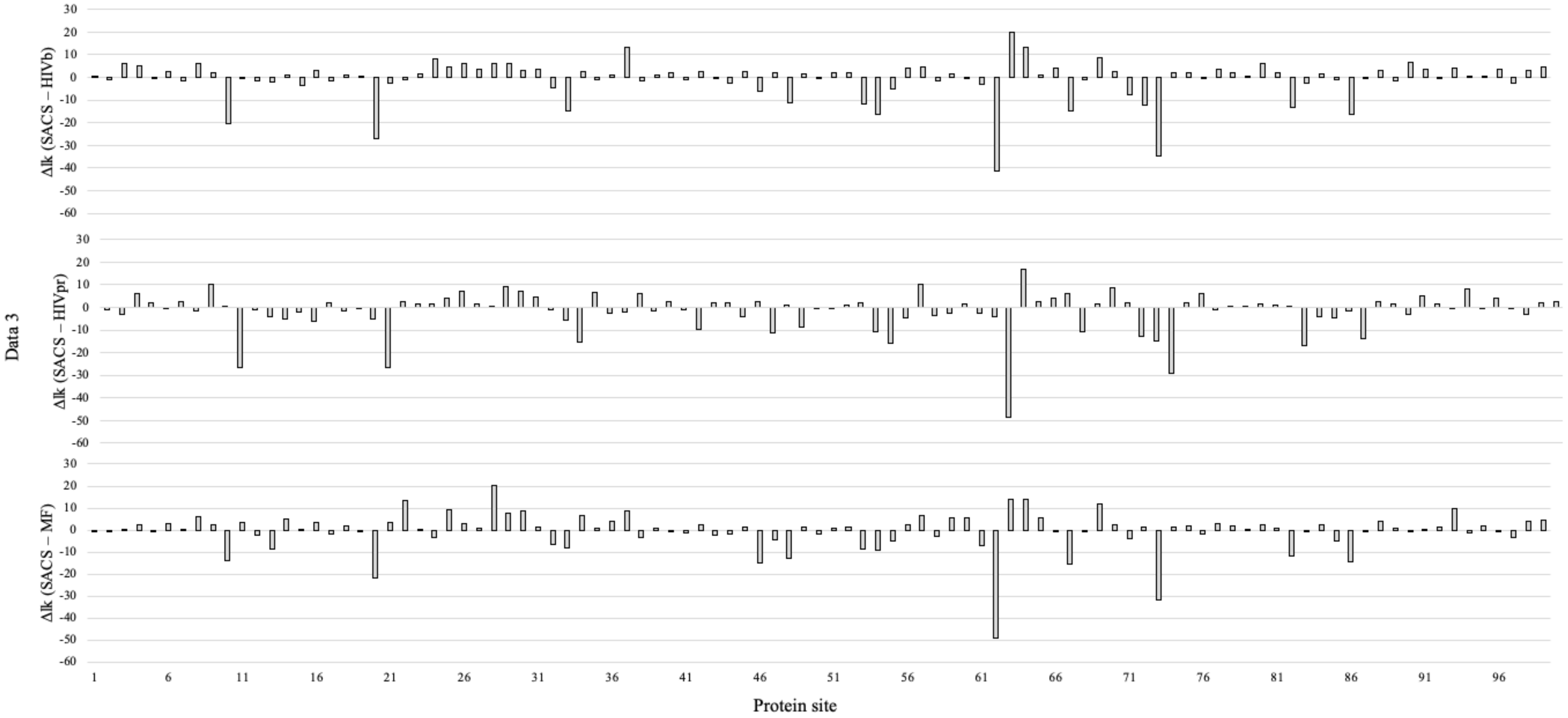
Data 3
Δlk (SACS – HIVb)
Δlk (SACS – HIVpr)
Δlk (SACS – MF)
Protein site

Figure S13. **Site-specific fitting of the different substitution models with global substitution rate variation among sites according to a Gamma distribution in data with variable molecular diversity and through phylogenetic likelihood.** Difference of Bayesian information criterion (BIC) scores between the mean-field (MF) structurally constrained substitution model and the structure and activity constrained substitution (SACS) model (plots on the left), and between the HIVpr empirical substitution model and the SACS model (plots on the right), where all the models include variation of the global substitution rate among sites according to a Gamma distribution (+G). The analyses were performed for the data with high, intermediate and low sequence identity shown in rows A, B and C, respectively. Note that positive ΔBIC scores indicate a better fitting of the SACS model with the data.

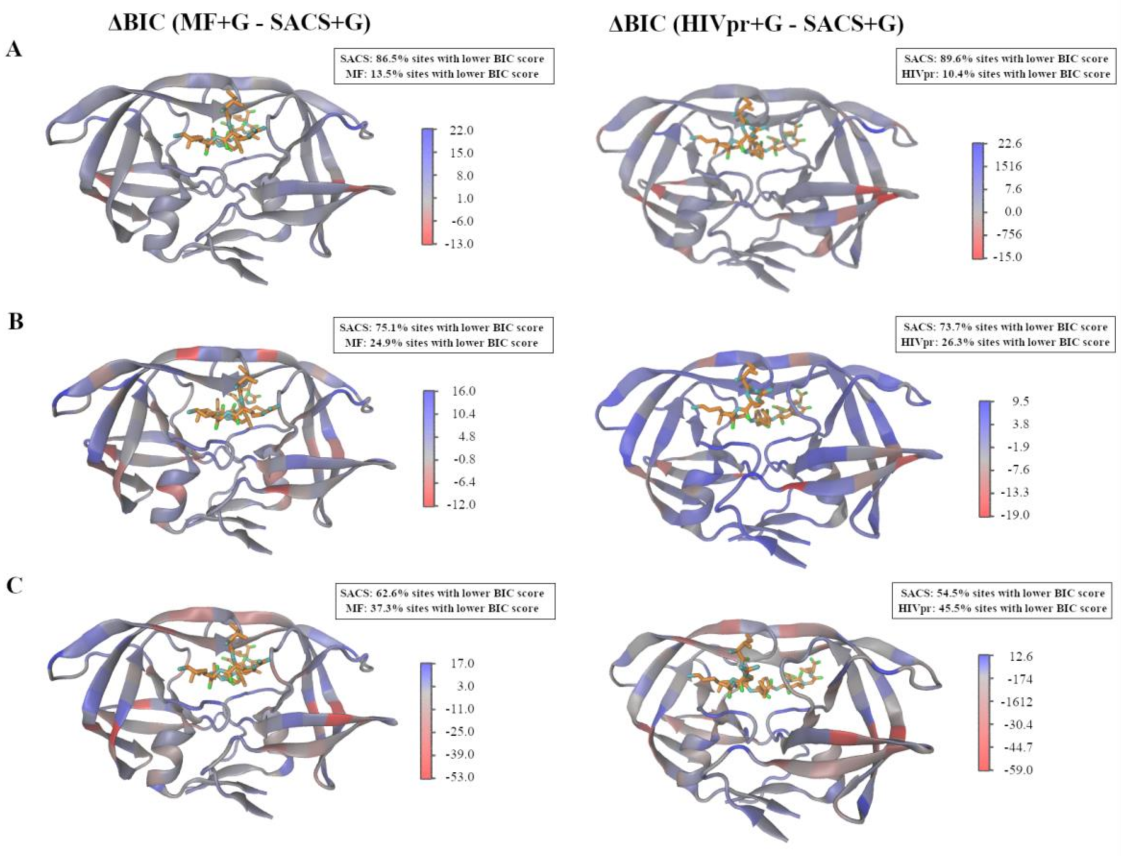

Figure S14. **Site-specific fitting of the HIVb empirical substitution model coonsdeirng and ignoring the global substitution rate variation among sites according to a Gamma distribution in data with variable molecular diversity and through phylogenetic likelihood.** Difference of Bayesian information criterion (BIC) scores between the HIVb empirical substitution model (that was selected as the best-fitting empirical substitution model for the corresponding data among the empirical substitution models available in *ModelTest-NG*) and activity constrained substitution (SACS) model (plots on the left), and between the HIVb empirical substitution model including variation of the global substitution rate among sites according to a Gamma distribution (+G) and activity constrained substitution (SACS) model (plots on the right). The analyses were performed for the data with high, intermediate and low sequence identity shown in rows A, B and C, respectively. Note that positive ΔBIC scores indicate a better fitting of the SACS model with the data.

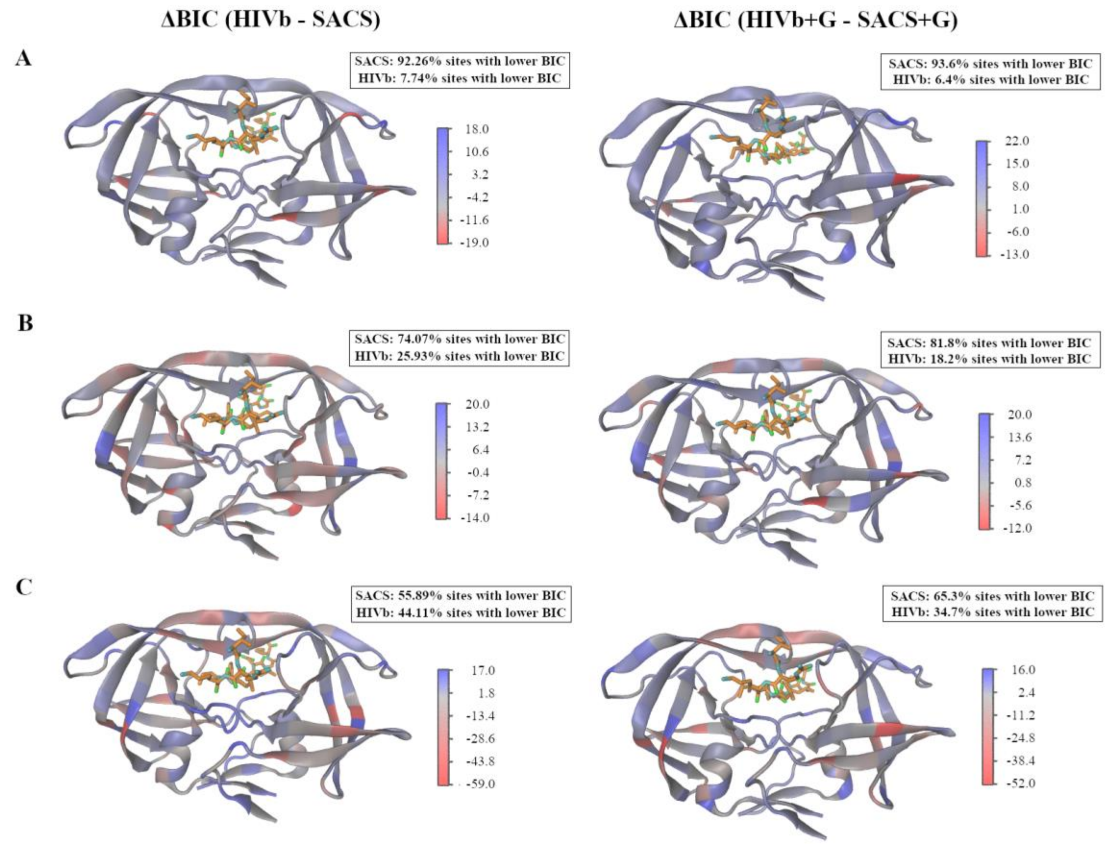

Figure S15. Site-specific effective number of amino acids in the additional test data and in the different substitution models of protein evolution. Site-specific effective number of amino acids obtained from all the additional test data (black line), the HIVb empirical substitution model (orange line), the HIVpr empirical substitution model (green line), the mean-field (MF) structurally constrained substitution model (gray line) and, the structure and activity constrained substitution (SACS) model (blue line). Note that the most realistic substitution model is that one producing site-specific effective number of amino acids closer to those observed in the real data. Site-specific effective number of amino acids for the test data are presented in Figure 6 (main text).

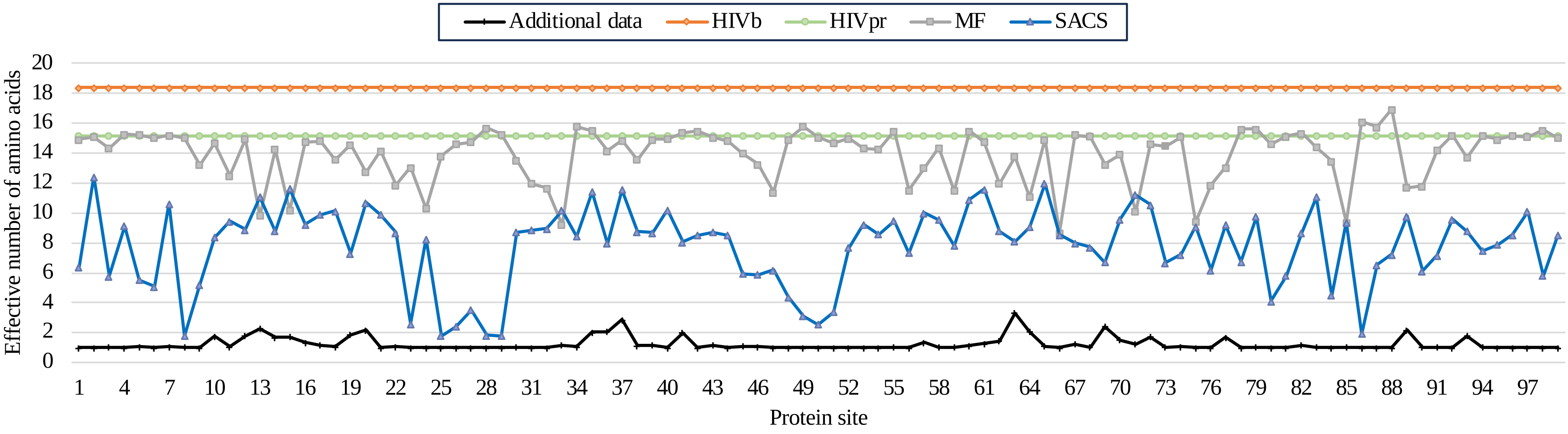

Supplement: msae026_Supplementary_Data [file msae026_supplementary_data.zip › SACS_SupMat_R2.pdf]
